# Tyrosine phosphorylation activates 6-phosphogluconate dehydrogenase and promotes tumor growth and radiation resistance

**DOI:** 10.1038/s41467-019-08921-8

**Published:** 2019-03-01

**Authors:** Ruilong Liu, Wenfeng Li, Bangbao Tao, Xiongjun Wang, Zhuo Yang, Yajuan Zhang, Chenyao Wang, Rongzhi Liu, Hong Gao, Ji Liang, Weiwei Yang

**Affiliations:** 1 State Key Laboratory of Cell Biology, CAS Center for Excellence in Molecular Cell Science, Innovation Center for Cell Signaling Network, Shanghai Institute of Biochemistry and Cell Biology, Chinese Academy of Sciences, University of Chinese Academy of Sciences, 200031 Shanghai, China; 2 Shanghai Key Laboratory of Molecular Andrology, Shanghai Institute of Biochemistry and Cell Biology, Chinese Academy of Sciences, University of Chinese Academy of Sciences, 200031 Shanghai, China; 3 Department of Radiation Oncology, First Affiliated Hospital of Wenzhou Medical College, Wenzhou, 325000 Zhejiang, China; 4 Department of Neurosurgery, XinHua Hospital School of Medicine, Shanghai Jiaotong University, 200092 Shanghai, China; 5 Precise Genome Engineering Center, School of Life Sciences, Guangzhou University, 510006 Guangzhou, China; 6 Chemical Biology Core Facility, Institute of Biochemistry and Cell Biology, Shanghai Institutes for Biological Sciences, Chinese Academy of Sciences, 200031 Shanghai, China; 7 Department of Immunology, Lerner Research Institute, Cleveland Clinic, Cleveland, OH 441195 USA; 8 Center for Medical Device Evaluation, China Drug Administration, State Administration for Market Regulation, 100081 Beijing, China

## Abstract

6-Phosphogluconate dehydrogenase (6PGD) is a key enzyme that converts 6-phosphogluconate into ribulose-5-phosphate with NADP^+^ as cofactor in the pentose phosphate pathway (PPP). 6PGD is commonly upregulated and plays important roles in many human cancers, while the mechanism underlying such roles of 6PGD remains elusive. Here we show that upon EGFR activation, 6PGD is phosphorylated at tyrosine (Y) 481 by Src family kinase Fyn. This phosphorylation enhances 6PGD activity by increasing its binding affinity to NADP^+^ and therefore activates the PPP for NADPH and ribose-5-phosphate, which consequently detoxifies intracellular reactive oxygen species (ROS) and accelerates DNA synthesis. Abrogating 6PGD Y481 phosphorylation (pY481) dramatically attenuates EGF-promoted glioma cell proliferation, tumor growth and resistance to ionizing radiation. In addition, 6PGD pY481 is associated with Fyn expression, the malignancy and prognosis of human glioblastoma. These findings establish a critical role of Fyn-dependent 6PGD phosphorylation in EGF-promoted tumor growth and radiation resistance.

## Abstract (web summary)

6-phosphogluconate dehydrogenase is commonly upregulated in cancers. Here, the authors show that activation of EGFR induces phosphorylation of this enzyme at Y481 to activate the pentose phosphate pathway, which consequently reduces ROS and accelerates DNA synthesis to promote tumor growth and radioresistance.

## Introduction

The reprogramming of cellular metabolism commonly exists in many types of cancer cells^1^. These aberrant alterations in metabolism provide both excessive energy and metabolic intermediates that are necessary for the rapid growth of cancer cells^2^. Aerobic glycolysis, also known as the Warburg effect, is a typical example: even in the presence of ample oxygen, rather than taking advantage of mitochondrial oxidative phosphorylation, most cancer cells rely more on glycolysis to produce adenosine 5’-triphosphate (ATP) and metabolic intermediates for biosynthesis of macromolecules and subsequent cell proliferation^3^.

Enhanced aerobic glycolysis in transformed cells provides more intermediates to be utilized in glycolytic shunts^4^. For instance, glucose-6-phoshate (G-6-P), derived from glycolysis, enters the pentose phosphate pathway (PPP), which generates nicotinamide adenine dinucleotide phosphate (NADPH) and ribose-5-phosphate (R-5-P)^4^. In normal conditions, >80% of total cytosolic NADPH is used for biosynthesis, with most of these NADPH consumed by fatty acid synthesis^5^. NADPH is also a crucial antioxidant. In contrast, it can also be used to produce glutathione (GSH), which in turn eliminates reactive oxygen species (ROS) that is produced during cell proliferation and generated by other stimuli, such as ionizing radiation (IR) and radical-generating compounds^6,7^. Another product R-5-P is a precursor for de novo, as well as salvage pathway of nucleic acid biogenesis that is important for mitosis and DNA repair^8^.

6-Phosphogluconate dehydrogenase (6PGD) is the third enzyme of the PPP that catalyzes the oxidative decarboxylation of 6-phosphogluconate (6-PG) to ribulose-5-phosphate (Ru-5-P) with concomitant reduction of NADP^+^ to NADPH. This protein often functions as a homodimer^9^. Accumulating data suggest that 6PGD is hyperactive in different types of cancer cells and plays a fundamental role in tumor growth^10–13^. In lung cancer cells, depletion of 6PGD leads to accumulation of p53 and subsequent cell senescence^13^. 6PGD can also be acetylated in lung cancer cells, which activates 6PGD to produce NADPH and Ru-5-P, thereby promoting lipids and RNA synthesis and reducing ROS levels^14^. Moreover, Ru-5-P, generated by 6PGD, inhibits 5' adenosine monophosphate-activated protein kinase (AMPK) activity to promote fatty acid synthesis by disrupting upstream LKB1 complex^15^. However, whether 6PGD can be phosphorylated and how this phosphorylation contributes to cancer progression remains unknown.

The epidermal growth factor receptor (EGFR) is frequently overexpressed in approximately 40% of glioblastoma (GBM). In approximately 50% of tumors with EGFR amplification, a specific EGFR mutant (EGFRvIII) can be detected. This mutant, which is generated from a deletion of exons 2–7 of the receptor, is constitutively active and highly oncogenic^16^. Substantial evidence suggests that EGFR plays a causal role in GBM pathogenesis and resistance to treatment^16,17^. However, how EGFR signaling reprograms cell metabolism to support GBM progression, especially the resistance to treatment, remains unclear.

In this study, we investigate the role of 6PGD phosphorylation in EGFR-promoted tumor growth and radiation resistance, highlighting the fundamental role of Fyn-dependent 6PGD phosphorylation in brain tumor development.

## Results

### 6PGD pY481 is required for EGF-enhanced 6PGD activity

**Fig. 1:**
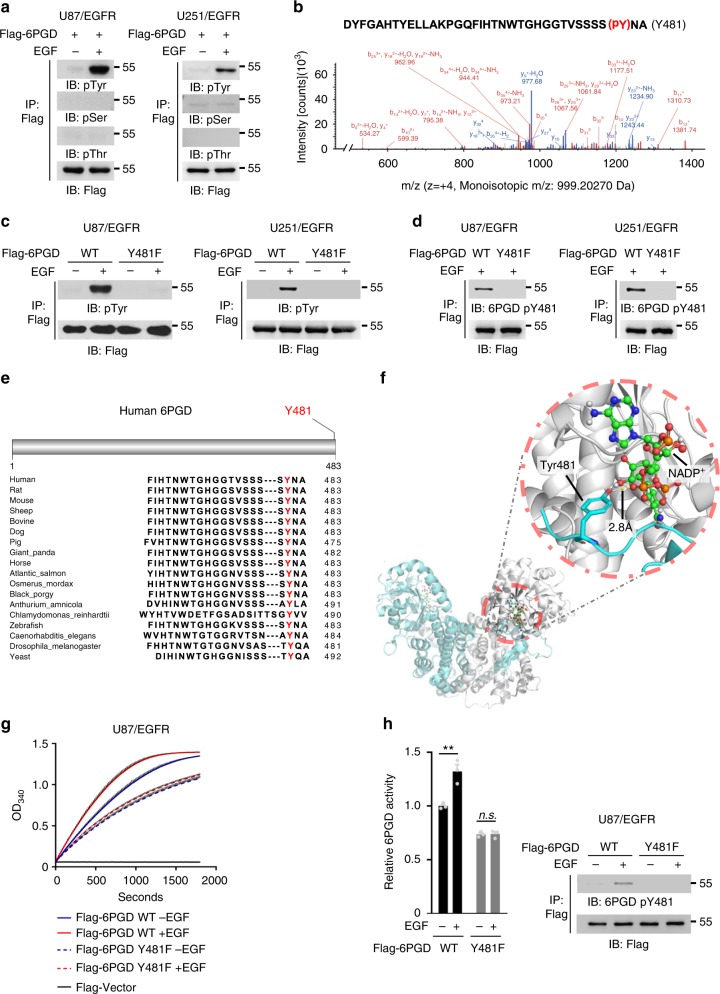
6-Phosphogluconate dehydrogenase (6PGD) pY481 is required for EGF-enhanced 6PGD activity. **a** U87/epidermal growth factor receptor (EGFR) cells (left panel) or U251/EGFR cells (right panel) stably expressing Flag-6PGD were treated with or without EGF (100 ng ml^-1^) for 30 min. pTyr phospho-tyrosine, pSer phospho-serine, pThr phospho-threonine. **b** Immunoprecipitated Flag-6PGD from U87/EGFR cells treated with or without EGF (100 ng ml^-1^) for 30 min, was subjected to mass spectrometry analyses. Mass spectrometry analysis of a tryptic fragment at m/z 999.20270 (*z* = + 4), matched to the charged peptide 1-DYFGAHTYELLAKPGQFIHTNWTGHGGTVSSSS(pY)NA-36. The probability of pY481 was 99.99%. **c** U87/EGFR cells (left panel) or U251/EGFR cells (right panel) stably expressing Flag-6PGD WT or Y481F were treated with or without EGF (100 ng ml^-1^) for 30 min. **d** U87/EGFR cells (left panel) or U251/EGFR cells (right panel) stably expressing Flag-6PGD WT or Y481F were treated with EGF (100 ng ml^-1^) for 30 min. **e** Sequence alignment of phosphorylated peptides among indicated species. **f** Representative image of structure of human 6PGD bound to NADP^+^ (PDB ID:2JKV) was shown. Dimeric 6PGD was shown as ribbons and NADP^+^ was shown as balls and sticks. 6PGD Y481 was shown as sticks. Cyan ribbon or gray ribbon represented two monomers in dimeric 6PGD, respectively. **g**, **h** Flag-6PGD WT or Y481F were immunoprecipitated from U87/EGFR cells with or without EGF (100 ng ml^-1^) treatment for 30 min. The enzymatic activity of immunoprecipitated Flag-6PGD proteins was examined (**g**). Statistical analyses of 6PGD activities (**h**). The activity of 6PGD was normalized against protein amounts. 6PGD WT activity without EGF treatment was normalized to 1.0. Data represent the mean ± SD of three independent experiments. Student’s *t*-test (unpaired, two tailed), **p < 0.01; n.s. not significant. Immunoprecipitation and immunoblotting analyses were performed with the indicated antibodies. Data are representative of at least three independent experiments. **a**, **c**, **d**, **h** Source data are provided as a Source Data file

To test whether 6PGD is phosphorylated upon EGFR activation, we generated U87 or U251 glioma cells stably expressing EGFR (U87/EGFR or U251/EGFR), and infected these cells and human primary GSC11 GBM cells with the lentivirus expressing Flag-tagged 6PGD (Flag-6PGD). Immunoblotting analyses of immunoprecipitated Flag-6PGD with anti-phospho-serine, anti-phospho-threonine, or anti-phospho-tyrosine antibodies showed that 6PGD was phosphorylated at tyrosine, but not at serine or threonine, upon EGFR activation (Fig. 1a). Mass spectrometry analyses of immunoprecipitated Flag-6PGD from U87/EGFR cells with or without EGF treatment showed that 6PGD was phosphorylated at tyrosine (Y) 481 after EGF treatment (Fig. 1b, Supplementary Fig. 1a). Mutation of Y481 into phenylalanine (F) almost completely blocked EGF-induced tyrosine phosphorylation of 6PGD, suggesting that Y481 is the major phosphorylated tyrosine in 6PGD (Fig. 1c, Supplementary Fig. 1b). This result was further supported by immunoblotting analyses with a custom-designed anti-phospho-6PGD Y481 (anti-6PGD pY481) antibody (Fig. 1d, Supplementary Fig. 1c). Sequence alignment analyses among various species showed that Y481 of 6PGD was highly conserved from yeast to human (Fig. 1e, Supplementary Fig. 1d). We further analyzed 6PGD protein structure and found that Y481, located in the dimeric interface of 6PGD, is close to the binding site of cofactor NADP^+^ (Fig. 1f). So, we next tested whether Y481 phosphorylation regulates 6PGD enzymatic activity. Flag-6PGD proteins were immunoprecipitated from U87/EGFR, U251/EGFR, or GSC11 cells stably expressing Flag-6PGD WT or Flag-6PGD Y481F with or without EGF treatment for 6PGD activity assays, which showed that EGF treatment increased the enzymatic activity of 6PGD WT, but failed to increase that of 6PGD Y481F (Figs. 1g, h, Supplementary Fig. 1e–h). Collectively, these results indicate that Y481 phosphorylation is essential for EGFR activation-enhanced 6PGD activity.

### Fyn phosphorylates 6PGD at Y481

**Fig. 2:**
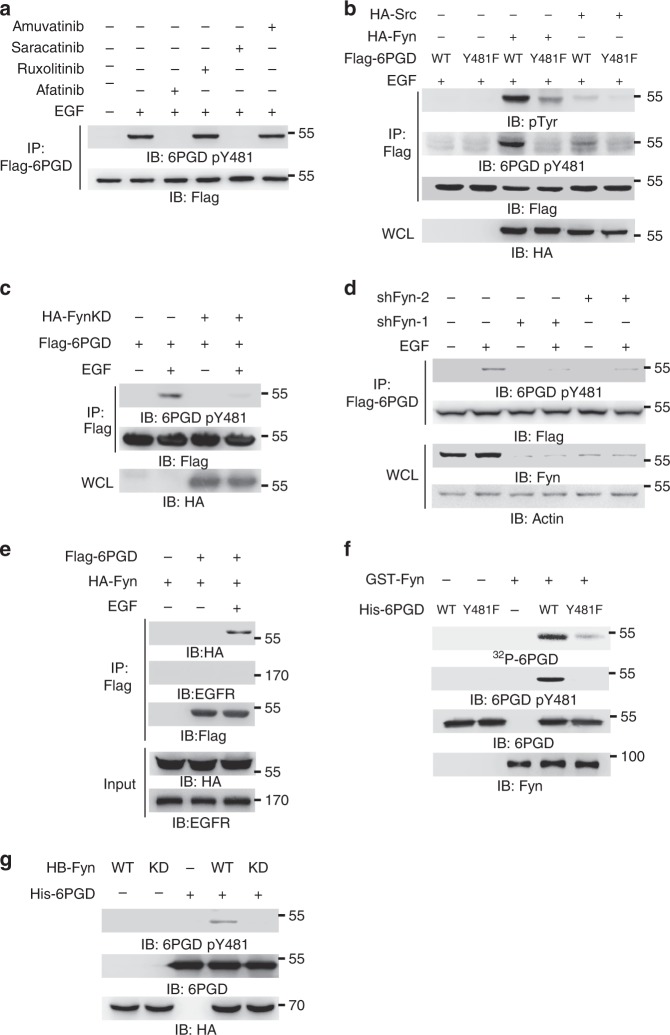
Fyn phosphorylates 6-phosphogluconate dehydrogenase (6PGD) at Y481. **a** HEK293T/epidermal growth factor receptor (EGFR) cells were pretreated with Afatinib (1 μM), Ruxolitinib (1 μM), Saracatinib (1 μM), or Amuvatinib (10 μM) for 3 h and then treated with EGF (100 ng ml^-1^) for 30 min. **b** HEK293T/EGFR cells stably expressing Flag-6PGD WT or Y481F were transiently co-transfected with or without HA-Src, HA-Fyn as indicated. **c** HEK293T/EGFR cells were transiently co-transfected with or without Flag-6PGD and a kinase-dead HA-Fyn (KD) mutant as indicated. Cells were treated with or without EGF (100 ng ml^-1^) for 30 min. **d** U87/EGFR cells were infected with the lentivirus expressing shNT or two shRNA sequences against Fyn (shFyn-1 and shFyn-2). These cells were then treated with or without EGF (100 ng ml^-1^) for 30 min as indicated. **e** U87/EGFR cells were transiently co-transfected with HA-Fyn and Flag-6PGD as indicated. Cells were treated with or without EGF (100 ng ml^-1^) for 30 min. **f** In vitro kinase assays were carried out with bacterial purified recombinant His-6PGD WT or Y481F and commercial purchased recombinant GST-Fyn. **g** HA-SBP (HB) tagged Fyn WT or kinase-dead HB-Fyn (KD) mutant was immunoprecipitated from U87/EGFR cells pretreated with EGF (100 ng ml^-1^) for 30 min and then used for in vitro kinase assays with bacterial purified His-6PGD. Immunoprecipitation and immunoblotting analyses were performed with the indicated antibodies. Data are representative of at least three independent experiments. Source data are provided as a Source Data file

To identify the kinase that phosphorylates 6PGD Y481 upon EGFR activation, we generated HEK293T cells stably expressing EGFR (HEK293T/EGFR), and transiently transfected HEK293T/EGFR or stably infected GSC11 cells with Flag-6PGD in the absence or presence of tyrosine kinase inhibitors, such as Afatinib (EGFR inhibitor), Ruxolitinib (JAK family inhibitor), Saracatinib (Src family inhibitor), and Amuvatinib (c-Kit inhibitor), respectively (Supplementary Fig. 2a). Immunoblotting analyses showed that the inhibition of EGFR or Src family abrogates EGF-induced 6PGD Y481 phosphorylation (Fig. 2a, Supplementary Fig. 2b). Src kinase family, a family of non-receptor tyrosine kinase, is the classical kinase family downstream of EGFR signaling. It includes nine members, such as Src, Yes, Fyn, Fgr, Lck, Hck, Blk, Lyn, and Frk. It has been shown that Fyn and Src are effectors of oncogenic EGFR signaling in GBM patients^18^. So, we overexpressed HA-Src or HA-Fyn in HEK293T/EGFR cells stably expressing Flag-6PGD. Immunoblotting analyses of immunoprecipitated Flag-6PGD with anti-phospho-tyrosine and anti-6PGD pY481 antibodies showed that Fyn, but not Src, phosphorylated 6PGD Y481 (Fig. 2b). Overexpression of a kinase-dead Fyn mutant (Fyn KD) in HEK293T/EGFR and GSC11 cells dramatically inhibited EGF-induced 6PGD Y481 phosphorylation in (Fig. 2c, Supplementary Fig. 2c). Furthermore, knockdown of Fyn using short hairpin RNA (shRNA) almost completely abrogated 6PGD Y481 phosphorylation after EGF stimulation in U87/EGFR cells (Fig. 2d), U251/EGFR cells (Supplementary Fig. 2d), GSC11 cells (Supplementary Fig. 2e), and HEK293T/EGFR cells (Supplementary Fig. 2f). These results indicate that Fyn is required for 6PGD Y481 phosphorylation upon EGFR activation.

We next examined the interaction between 6PGD and Fyn or EGFR. Coimmunoprecipitation assays showed that 6PGD interacted with Fyn but not with EGFR after EGF treatment (Fig. 2e). To determine whether Fyn directly phosphorylates 6PGD, we carried out in vitro kinase assays by mixing purified recombinant His-6PGD and commercial purchased recombinant Glutathione S-transferase (GST)-Fyn. The experiment showed that Fyn phosphorylated 6PGD and this phosphorylation was dramatically reduced by Y481F mutation. This 6PGD Y481 phosphorylation was further validated by the immunoblotting analyses using anti-6PGD pY481 antibody (Fig. 2f). We also examined whether EGFR or Src could phosphorylate 6PGD by mixing His-6PGD and purchased recombinant GST-Src or GST-EGFR. The in vitro kinase assays showed that neither Src nor EGFR could directly phosphorylate 6PGD. The autophosphorylation of kinase suggested both GST-Src and GST-EGFR were active (Supplementary Fig. 2g, h). To determine whether Fyn kinase activity is required for 6PGD phosphorylation, HA-streptavidin binding peptide (HB) tagged Fyn WT or kinase-dead HB-Fyn KD mutant precipitated from EGF-treated U87/EGFR cells was mixed together with recombinant His-6PGD for the in vitro kinase assay. The experiment showed that Fyn WT phosphorylated 6PGD Y481, whereas Fyn KD failed to do so (Fig. 2g). These results indicate that Fyn is the kinase that directly phosphorylates 6PGD at Y481.

### 6PGD pY481 enhances NADP^+^ binding affinity of 6PGD

We next investigated how Y481 phosphorylation promotes 6PGD enzymatic activity. As shown in Fig. 1f, Y481 is located in dimeric interface and close to the binding site of NADP^+^ (2.8 Å). We thus hypothesized that Y481 phosphorylation likely activates 6PGD by either fostering its dimerization or increasing its NADP^+^ affinity. We first tested whether EGF stimulation regulates 6PGD dimer formation in U87/EGFR cells co-transfected with Flag-6PGD and HA-6PGD in the absence or presence of EGF treatment. Coimmunoprecipitation analyses showed that EGF treatment did not change the amount of HA-6PGD precipitated by Flag-6PGD, suggesting that EGF does not regulate 6PGD dimerization (Supplementary Fig. 3a). This result was further supported by protein cross-linking assays, showing that EGF treatment did not affect dimer/monomer ratio of 6PGD (Supplementary Fig. 3b).

**Fig. 3:**
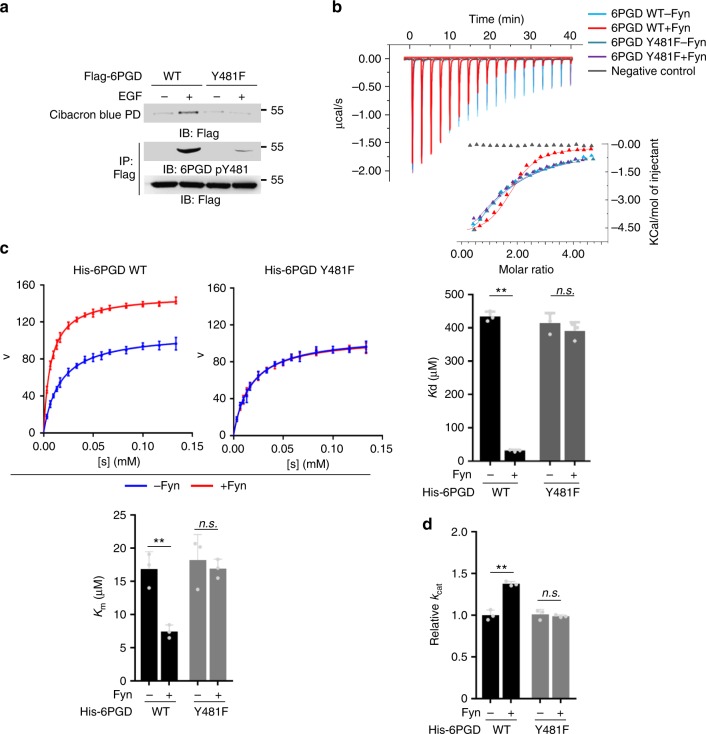
6-Phosphogluconate dehydrogenase (6PGD) pY481 enhances NADP^+^ binding affinity of 6PGD. **a** U87/epidermal growth factor receptor (EGFR) cells stably expressing Flag-6PGD WT or Y481F were treated with or without EGF (100 ng ml^-1^) for 30 min. Cell lysates were incubated with Cibacron blue beads mimicking NADP^+^ for a pulldown assay. **b** In vitro kinase assays were performed by incubating bacterial purified recombinant His-6PGD WT or Y481F with or without active recombinant Fyn. ITC assays were performed with pulldown 6PGD variants (0.05 mM) and NADP^+^ (1 mM) (top panel). His peptides were used as negative control. Statistical analyses of *K*d values of 6PGD variants for NADP^+^ were presented (bottom panel). **c**, **d** In vitro kinase assays were performed by incubating bacterial purified recombinant His-6PGD WT or Y481F with or without recombinant active Fyn. *K*_m_ (**c**) and *k*_cat_ (**d**) of 6PGD variants were determined. Immunoprecipitation and immunoblotting analyses were performed with the indicated antibodies. Data are representative of at least three independent experiments. **b**–**d** Data represent the mean ± SD of three independent experiments. Student’s *t*-test (unpaired, two tailed), **p < 0.01; n.s. not significant. Source data are provided as a Source Data file

Cibacron blue is a NAD^+^/NADP^+^ mimic compound, widely used to examine the binding affinity of metabolic enzymes to NAD^+^/NADP^+^^19^. So, we performed pulldown assays using Cibacron blue beads and found that EGF treatment increased the binding of 6PGD WT to NADP^+^, but failed to increase the binding of 6PGD Y481F to NADP^+^ (Fig. 3a). Moreover, this result was also supported by isothermal titration calorimetry (ITC) assays with bacterial purified recombinant 6PGD WT or Y481F in the absence or presence of Fyn, which showed that Fyn that phosphorylates 6PGD Y481 dramatically enhanced the NADP^+^ affinity of 6PGD WT while Y481F mutation completely abrogated such function of Fyn (Fig. 3b, Supplementary Fig. 3c). Similar results were obtained in the ITC assays with immunoprecipitated 6PGD variants from HEK293T/EGFR cells expressing Flag-6PGD WT or Y481F with or without EGF treatment, which showed that EGF increased the NADP^+^ affinity of 6PGD WT while Y481F mutation blocked such increased NADP^+^ affinity (Supplementary Fig. 3c, d).

To further characterize the enzyme kinetics of 6PGD regulated by Y481 phosphorylation, we determined the Michaelis constant (*K*_m_) and the turnover rate (*k*_cat_) of purified recombinant 6PGD with or without Y481 phosphorylation. The data showed that Fyn greatly decreased *K*_m_ value of 6PGD WT while Y481F mutation abrogated such function of Fyn (Fig. 3c, Supplementary Fig. 3e), which is consistent with the ITC data of purified 6PGD. In addition, Fyn also promoted the *k*_cat_ of 6PGD WT but failed to do so with 6PGD Y481F (Fig. 3d). Collectively, these results show that Y481 phosphorylation promotes 6PGD activity through increasing both NADP^+^ affinity and turnover rate of 6PGD.

### 6PGD pY481 promotes DNA synthesis and glioma progression

**Fig. 4:**
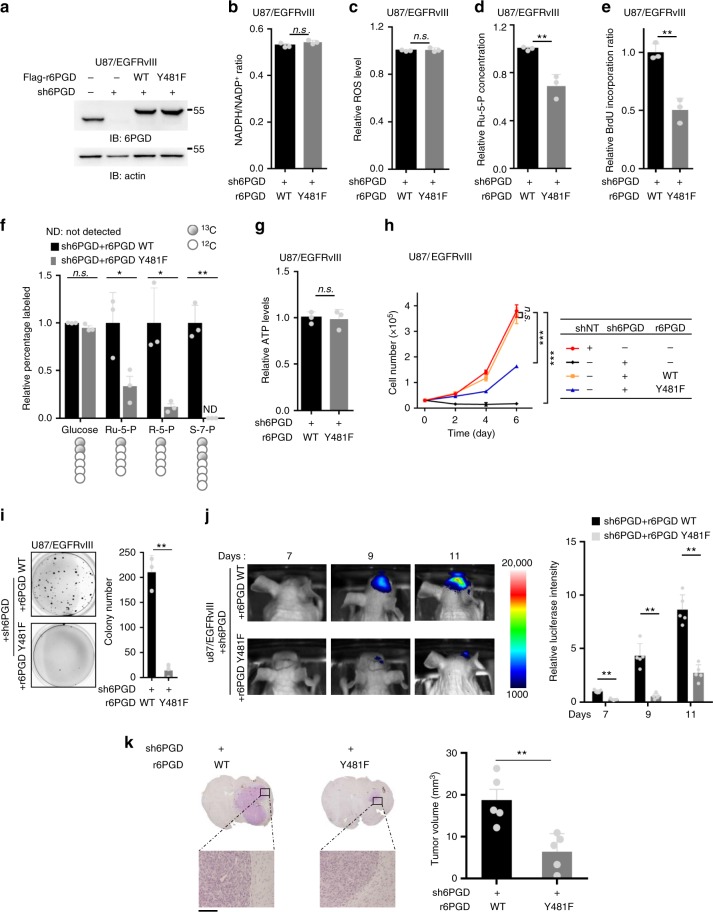
6-Phosphogluconate dehydrogenase (6PGD) Y481 phosphorylation promotes DNA synthesis and glioma progression. **a** U87/EGFRvIII cells stably expressing luciferase were depleted of endogenous 6PGD and reconstituted with the expression of r6PGD WT or Y481F. The expression of 6PGD was examined using immunoblotting assays. **b**–**e** NADP^+^/NADPH ratio (**b**), relative reactive oxygen species (ROS) levels (**c**), cellular ribulose-5-phosphate (Ru-5-P) level (**d**), and BrdU incorporation ratio (**e**) were measured in these cells generated in **a**. **f** The flux through the pentose phosphate pathway (PPP) was determined using d-glucose-1,2-^13^C_2_ in these cells generated in **a**. **g** Cellular ATP levels were determined in these cells generated in **a**. **h**, **i** Cell proliferation (**h**) and colony formation (**i**) were determined in these cells generated in **a**. **j**, **k** These cells generated in **a** (2 × 10^5^ per mouse) were intracranially injected into randomized athymic nude mice (five mice per group). Bioluminescence imaging of tumor growth were carried out. Real time images were presented (**j**, left panel) and the intensities of luciferase were quantified (**j**, right panel). After 11 days, tumor growth was examined. Hematoxylin and eosin (H&E)-stained coronal brain sections show representative tumor xenografts. Scale bar, 100 μm (**k**, left panel). Representative images of tumor boundaries were presented with ×200 magnification. Tumor volumes were measured using length (***a***) and width (***b***) and calculated using the equation: *V* = *ab*^2^/2 (**k**, right panel). Data represent the mean ± SD of luciferase intensity of five mice per group. Student’s *t-*test (unpaired, two tailed), **p < 0.01. **b**–**i** Data represent the mean ± SD of three independent experiments. Student’s *t-*test (unpaired, two tailed), **p < 0.01; n.s. not significant. Source data are provided as a Source Data file

To determine the effects of 6PGD Y481 phosphorylation on PPP, we depleted endogenous 6PGD in U87 or U251 cells stably expressing constitutively active EGFRvIII mutant (U87/EGFRvIII or U251/EGFRvIII) or GSC11 cells and rescued these cells with shRNA-resistant (r) Flag-6PGD WT or Y481F mutant (Fig. 4a, Supplementary Fig. 4a). As shown in Fig. 4b and Supplementary Fig. 4b, 6PGD Y481F mutation did not affect NADPH/NADP^+^ ratio in tumor cells. As NADPH is a crucial antioxidant, we next examined the intracellular ROS levels in these tumor cells, which showed that tumor cells rescued with r6PGD WT had similar intracellular ROS levels with the cells rescued with r6PGD Y481F (Fig. 4c, Supplementary Fig. 4c). In contrast, tumor cells rescued with r6PGD Y481F had much lower intracellular Ru-5-P (the product of 6PGD) than the cells rescued with r6PGD WT (Fig. 4d, Supplementary Fig. 4d). R-5-P, one of the end products of PPP, can be used in the synthesis of nucleotides and nucleic acids. So, we next examined DNA synthesis of tumor cells using BrdU incorporation assay. The experiment showed that tumor cells rescued with r6PGD Y481F had much less DNA synthesis than the cells rescued with r6PGD WT (Fig. 4e, Supplementary Fig. 4e). Furthermore, we examined the flux through the PPP using d-glucose-1,2-^13^C_2_ in 6PGD-depleted U87/EGFRvIII cells rescued with r6PGD WT or Y481F, which indicated that rescued expression of r6PGD Y481F dramatically reduced the flux through the PPP (Fig. 4f). In addition, we examined the intracellular ATP levels in those genetically modified U87, U251, or GSC11 cells, and found no difference between tumor cells rescued with r6PGD WT and the cells rescued with r6PGD Y481F (Fig. 4g, Supplementary Fig. 4f).

We next investigated the biological function of 6PGD Y481 phosphorylation. Cell proliferation assays showed that 6PGD knockdown greatly inhibited cell proliferation of U87/EGFRvIII, which were completely restored by the rescued expression of r6PGD WT, but only partially (~50%) restored by that of r6PGD Y481F (Fig. 4h). Similarly, rescued expression of r6PGD Y481F dramatically attenuated the proliferation of U251/EGFRvIII or GSC11 cells compared with that of r6PGD WT (Supplementary Fig. 4g). In addition, colony formation assays also showed that U87/EGFRvIII cells rescued with r6PGD Y481F had much less colonies formation than the cells rescued with r6PGD WT (Fig. 4i).

To determine the role of 6PGD Y481 phosphorylation in tumorigenesis, we intracranially injected endogenous 6PGD-depleted U87/EGFRvIII cells with rescued expression of r6PGD WT or Y481F into randomized athymic nude mice. Bioluminescence imaging of mice showed that tumor cells rescued with r6PGD WT elicited a rapid tumor growth, whereas the cells rescued with the expression of r6PGD Y481F had much slower tumor growth (Fig. 4j). Consistently, dissected mouse brain sections on 11 days after the inoculation showed that much smaller tumors were observed in the mice injected with the cells rescued with r6PGD Y481F than those in the mice injected with the cells rescued with r6PGD WT (Fig. 4k). Taken together, these results show that 6PGD Y481 phosphorylation is required for DNA synthesis, glioma cell proliferation, and brain tumor development.

### 6PGD pY481 enhances the resistance of tumor cells to IR

**Fig. 5:**
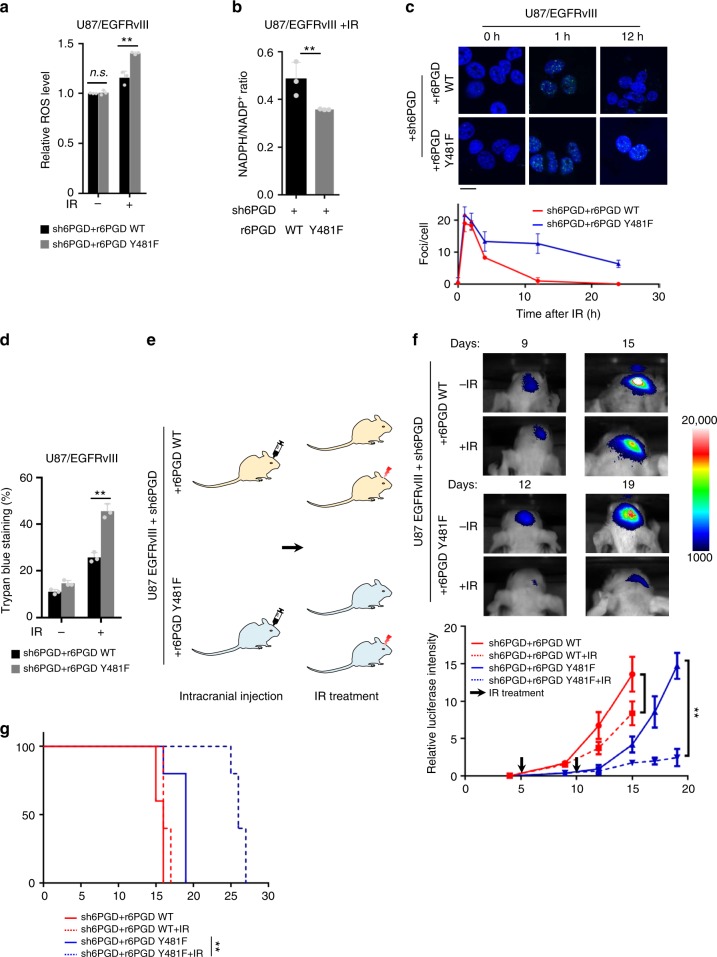
6-Phosphogluconate dehydrogenase (6PGD) pY481 enhances the resistance of tumor cells to ionizing radiation (IR). **a**, **b** U87/EGFRvIII cells stably expressing luciferase were depleted of endogenous 6PGD and reconstituted with the expression of r6PGD WT or Y481F. These cells were treated with or without X-ray radiation (10 Gy) and cultured for 8 h. Reactive oxygen species (ROS) levels were measured using ROS assay kit (**a**). NADPH/NADP^+^ ratio was determined using NADP^+^/NADPH quantitation kit (**b**). Data represent the mean ± SD of three independent experiments. Student’s *t*-test (unpaired, two tailed), **p < 0.01; n.s. not significant. **c** These cells generated in **a** were subjected to X-ray radiation (2 Gy) and cultured for indicated times. Immunofluorescence staining was performed using anti-γH2AX antibody. Data represent the mean ± SD of three independent experiments. Scale bar, 20 μm. **d** These cells generated in **a** were subjected to X-ray radiation (10 Gy) and cultured for 48 h. Cell death was determined using Trypan blue staining. Data represent the mean ± SD of three independent experiments. Student’s *t*-test (unpaired, two tailed), **p < 0.01. **e**–**g** Schematic diagram of experimental procedure in which these genetically modified cells generated in **a** (1 × 10^5^ per mouse) were intracranially injected into randomized athymic nude mice (five mice per group) and then treated with or without IR (γ) radiation (4 Gy) (**e**). Bioluminescence imaging of tumor growth were carried out. Real time images were presented (**f**, top panel) and the intensities of luciferase were quantified (**f**, bottom panel) using Xenogen IVIS. Data represent the mean ± SD of luciferase intensity of five mice per group. Student’s *t-*test (unpaired, two tailed), **p < 0.01 Survival durations of these implanted mice were compared (**g**). Log-rank test, **p < 0.01. **a**–**d**, **f**, **g** Source data are provided as a Source Data file

IR is used extensively to treat many different types of human cancers, including glioma. IR induces the production of ROS, which play an important causative role in apoptotic cell death^20^. The PPP is central for the production of the reducing equivalents, NADPH that reduces glutathione via glutathione reductase, which converts reactive H_2_O_2_ to H_2_O by glutathione peroxidase^6^. So, we wondered whether EGF-induced 6PGD Y481 phosphorylation is involved in the resistance of tumor cells to IR. To test this hypothesis, we first examined ROS levels in 6PGD-depleted U87/EGFRvIII, U251/EGFRvIII, or GSC11 cells with rescued expression of r6PGD WT or Y481F in the absence or presence of IR treatment. The experiments showed that, before IR treatment, tumor cells rescued with r6PGD Y481F had similar ROS levels with the cells rescued with r6PGD WT. In contrast, after IR treatment, higher ROS levels were detected in the cells rescued with r6PGD Y481F than in the cells rescued with r6PGD WT (Fig. 5a, Supplementary Fig. 5a). We next examined the NADPH/NADP^+^ ratio in these cells after IR treatment. In line with ROS levels, NADPH/NADP^+^ ratio were much lower in the cells rescued with r6PGD Y481F than that in the cells rescued with r6PGD WT after IR treatment (Fig. 5b, Supplementary Fig. 5b). IR leads to DNA damage either through directly destroying atomic structure or via products of water radiolysis, such as ROS or reactive nitrogen species (NOS). So, we next examined DNA damage in these tumor cells after IR treatment using immunofluorescence staining with anti-rH2AX antibody. The experiment showed that the rate of DNA damage repair was much slower in the cells rescued with r6PGD Y481F than that in the cells rescued with r6PGD WT (Fig. 5c, Supplementary Fig. 5c). Consistently, cell viability assays showed that tumor cells rescued with r6PGD Y481F had much more cell death than the cells recued with r6PGD WT after IR treatment (Fig. 5d, Supplementary Fig. 5d).

In addition, we intracranially injected 6PGD-depleted U87/EGFRvIII cells with rescued expression of r6PGD WT or Y481F into randomized athymic nude mice followed by the treatment of IR (Fig. 5e). Bioluminescence imaging of mice showed that the tumors in the mice implanted with the cells rescued with r6PGD Y481F were more sensitive to IR treatment than the tumors in the mice implanted with the cells rescued with r6PGD WT (Fig. 5f). Those mice implanted with the cells rescued with r6PGD Y481F had much longer survival time after IR treatment than the mice implanted with the cells rescued with r6PGD WT (Fig. 5g). Collectively, these results indicate that 6PGD Y481 phosphorylation is required for ROS detoxification and tumor cell survival upon IR treatment.

### 6PGD pY481 correlates with the malignancy of human GBM

**Fig. 6:**
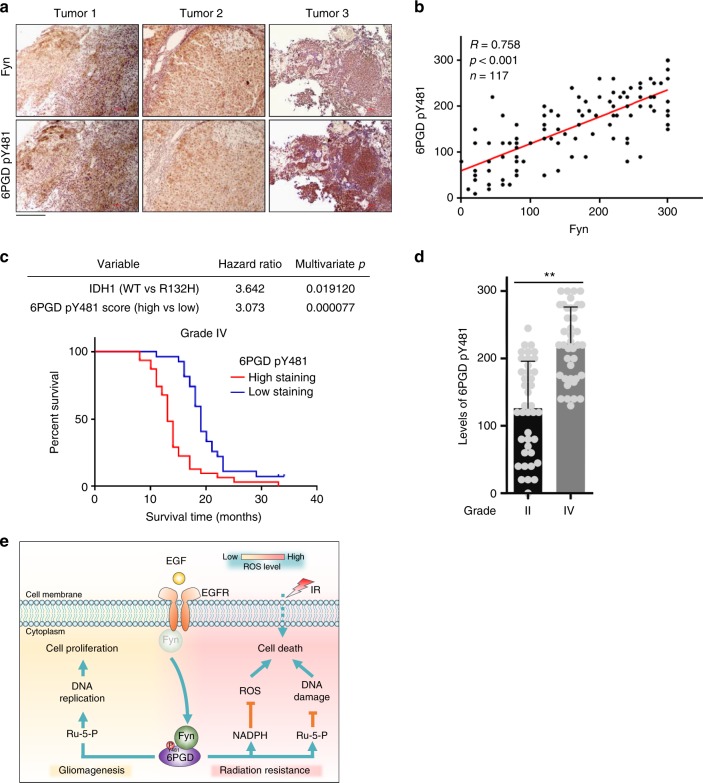
6-Phosphogluconate dehydrogenase (6PGD) pY481 correlates with the malignancy of human glioblastoma (GBM). **a**, **b** Immunohistochemical analyses of 117 specimens from glioma patients were performed using anti-6PGD pY481, anti-Fyn antibodies. Representative images of IHC staining of three glioma specimens were shown. Scale bar, 100 μm (**a**). Semiquantitative scoring (using a scale from 0 to 300) was carried out (**b**). Pearson’s correlation test, *p* < 0.001. **c** Survival durations of 58 GBM patients with low (0–170 staining scores, blue curve) versus high (175–300 staining scores, red curve) 6PGD pY481 levels (low, 26 patients; high, 32 patients) were compared. The table (top) shows the multivariate analysis, indicating the significance levels of the association of 6PGD Y481 phosphorylation (Cox regression, *p* = 0.000077) or IDH1 mutation (Cox regression, *p* = 0.019120) with patient survival. Landmark represents censored (alive at last clinical follow-up) patients. **d** Immunohistochemistry (IHC) staining was performed in 40 diffuse astrocytoma (WHO grade II) specimens and 40 GBM (WHO grade IV) specimens using anti-6PGD pY481 antibody. Staining scores of the diffuse astrocytoma (WHO grade II) specimens were compared with those of GBM specimens. Student’s *t*-test (unpaired, two tailed), **p < 0.01. **e** Schematic representation of 6PGD phosphorylation-promoted tumor growth and radiation resistance. Upon epidermal growth factor receptor (EGFR) activation, Fyn phosphorylates 6PGD Y481 and increases its enzymatic activity, which subsequently promotes more NADPH and ribulose-5-phosphate (Ru-5-P) production. Increased Ru-5-P facilitates DNA replication, thereby promoting tumor cell proliferation. Upon ionizing radiation (IR) stimulation, more NADPH and Ru-5-P detoxify increased reactive oxygen species (ROS) and boost DNA damage repair, thereby promoting tumor cell survival. **b**–**d** Source data are provided as a Source Data file

To define the clinical relevance of our finding that Fyn phosphorylates 6PGD Y481 upon EGFR activation, we performed immunohistochemistry (IHC) analyses in 117 glioma specimens using anti-6PGD pY481 and anti-Fyn antibodies. The antibody specificities were validated using IHC analyses of GBM specimens with specific blocking peptides or proteins (Supplementary Fig. 6a, b). The specificity of anti-6PGD pY481 was also validated by IHC analyses in 6PGD-depleted U87/EGFRvIII cells rescued with r6PGD WT or Y481F, which showed that this antibody was not able to recognize 6PGD Y481F (Supplementary Fig. 6c). As shown in Figs. 6a, b, the levels of 6PGD Y481 phosphorylation correlated with the levels of Fyn protein. Quantification of the staining on a scale of 0–300 showed the correlation between these two proteins was significant (*R* = 0.758).

The survival durations for the 58 GBM patients, all of whom received standard adjuvant radiotherapy after surgery, followed by treatment with an alkylating agent (temozolomide in the majority of cases), were compared between the patients with low 6PGD Y481 phosphorylation (0–170 staining) and the patients with high 6PGD Y481 phosphorylation (175–300 staining). Patients whose tumors had low levels of 6PGD Y481 phosphorylation had a median survival of 19 months, and patients whose tumors had high levels of 6PGD Y481 phosphorylation had a significantly lower median survival of 13 months (Fig. 6c). IDH mutation status is a fundamental division of gliomas and IDH mutant gliomas are fundamentally different from IDH wild-type gliomas, to the point where the WHO distinguishes them in the diagnostic criteria^21^. Thus, we next performed IHC analyses of IDH mutation using anti-IDH1 R132H antibody in these GBM specimens. The specificity of anti-IDH1 R132H was validated by the peptide blocking assay (Supplementary Fig. 6d). A multivariate analysis of these GBM patient survivals was carried out based on IHC score of 6PGD Y481 phosphorylation and IDH1 R132H mutation, which suggested that either 6PGD Y481 phosphorylation or IDH1 R132H mutation could be an independent predictor of GBM patient survival (Fig. 6c). In addition, we also compared the survival of GBM patients with wild-type IDH between the patients with low 6PGD pY481 scores and the patients with high 6PGD pY481 scores, which showed that 6PGD Y481 phosphorylation could also separate these patients into good- and poor-prognostic groups (Supplementary Fig. 6e). These results support a role for 6PGD Y481 phosphorylation in the clinical behavior of human GBM and reveal a relationship between 6PGD Y481 phosphorylation and clinical aggressiveness of the tumor.

To further explore this relationship, we examined whether the levels of 6PGD Y481 phosphorylation correlates with the grades of glioma malignancy. Levels of 6PGD Y481 phosphorylation in samples from patients (40 cases) with low-grade diffuse astrocytoma (WHO grade II; median survival time >5 years) were compared with those in samples from patients (40 cases) with high-grade GBM. IHC analyses showed that lower levels of 6PGD Y481 phosphorylation were present in low-grade tumors than in GBM specimens (Fig. 6d). Most low-grade astrocytomas (>80%) are IDH mutant, whereas most GBM (>90%) are IDH wild-type. We thus tested the possibility that the differences observed in 6PGD pY481 staining (Fig. 6d) is due to IDH mutation status, rather than grade. We performed IHC analyses of IDH mutation using anti-IDH1 R132H antibody in those samples from patients with low-grade diffuse astrocytoma or with high-grade GBM, respectively. The data showed that patients with IDH1 R132H mutation had similar levels of 6PGD Y481 phosphorylation with the patients without such IDH mutation no matter what grades the tumors were (Supplementary Fig. 6f).

## Discussion

It has been shown that 6PGD plays an important role in cancer progression, while the underlying mechanisms have not been fully understood. In this study, we demonstrate that Fyn phosphorylates 6PGD Y481 upon EGFR activation. Y481 phosphorylation enhances 6PGD activity by increasing its NADP^+^-binding affinity to activate the PPP for NADPH production and nucleotides synthesis, which subsequently promotes tumor cell proliferation and radiation resistance (Fig. 6e). These findings underscore the essential role of 6PGD phosphorylation in EGFR-promoted tumor growth and radiation resistance, suggesting therapeutic potential to dampen brain tumor development and sensitize glioma to radiotherapy through inhibiting 6PGD phosphorylation.

Metabolic enzymes are frequently regulated by various types of posttranslational modifications (PTMs), such as phosphorylation, acetylation, and ubiquitination. Among the PTMs, phosphorylation is especially important for protein function as this modification activates (or deactivates) almost half of the enzymes, thereby regulating their function^22,23^. In present study, we observe 6PGD phosphorylation for the first time, which responds to EGFR signaling and plays an important role in EGFR-promoted brain tumor development. This phosphorylation increases the binding of 6PGD to NADP^+^ to enhance enzymatic activity of 6PGD. 6PGD is also acetylated. Shan et al. report that acetyl-CoA acetyltransferase 2 (ACAT2) and dihydrolipoamide S-acetyltransferase (DLAT) acetylates 6PGD K76 and K294 to enhance 6PGD activity in lung cancer cells^14^. Altogether, these studies suggest that, in response to complex tumor microenvironment, 6PGD Y481 phosphorylation along with other PTMs regulates 6PGD activity and subsequently activate the PPP to maintain redox homeostasis and accelerate DNA synthesis, which ultimately enhances tumor cell proliferation and resistance to treatment.

In addition, IHC analyses of tumors from GBM patients indicate that 6PGD Y481 phosphorylation correlates with the malignancy and prognosis of GBM. This result implicates the prognostic potential of 6PGD phosphorylation in GBM.

The EGFR and the EGF family of peptide growth factor have a central role in the pathogenesis and progression of various carcinoma types^24^. It has been recently reported that EGFR signaling regulates tumor cell metabolism to promote tumor growth. For example, EGFR activation increases pyruvate kinase isozymes M2 (PKM2) expression by activating protein kinase C epsilon type/nuclear factor kappa-light-chain-enhancer of activated B cells (PKCε/NF-κB) signaling pathway, which therefore promotes glycolysis and subsequent tumor cell proliferation^25^. EGFRvIII expression upregulates heterogeneous nuclear ribonucleoprotein A1 splicing factor to increase glycolytic gene expression^26^. EGFR/phosphoinositide 3-kinases (PI3K)/Akt signaling also activates SREBP-1, which upregulates ACC, FAS, and SCD1 to promote de novo fatty acid synthesis^27^. We found here that EGFR activation can enhance 6PGD activity to activate the PPP, which produces NADPH for detoxification of intracellular ROS and R-5-P for nucleotides synthesis.

Although the activation of pro-proliferative and anti-apoptotic pathways by EGFR is an established concept, recent reports reveal a novel link between EGFR signaling and the repair of radiation-induced DNA double-strand breaks (DSBs). However, how EGFR signaling contributes to the radiation resistance of tumors remains elusive. In our study, we found that EGFR activation promotes Fyn-dependent 6PGD Y481 phosphorylation, which enhances 6PGD activity and activates pentose phosphate pathway to provide NADPH for ROS detoxification and nucleotides pool for DNA damage repair. Radiation-induced ionizations may act directly on the cellular component molecules (such as DNA) or indirectly on water molecules, causing water-derived radicals, including ROS or NOS^20^. Radicals react with nearby DNA in a very short time, resulting in breakage of chemical bonds or oxidation of the affected DNA. EGFR-activated PPP produces a large amount of NADPH that provides the reducing equivalents for oxidation reduction involved in protecting against ROS-caused DNA damage, allowing GSH generation. In addition, de novo nucleotide biosynthesis is often upregulated in cancer cells^28^, as sufficient nucleotide pools are required to maintain DNA fidelity during replication^29^ and to bypass oncogene-induced senescence^30^. Multiple levels of interaction between purine biosynthesis and DNA damage response have been discovered^31^. Upon EGFR activation, increased rate of PPP yields elevated levels of R-5-P and consequently 5-phosphoribosyl-1-pyrophosphate (PRPP). The increases in PRPP then result in excess purine biosynthesis that provides sufficient nucleotide pools to support DNA damage repair. Collectively, these results show that EGFR signaling activates PPP through 6PGD to enhance the radiation resistance of tumor cells and provide molecular basis to overcome GBM radioresistance by inhibiting 6PGD phosphorylation.

Fyn is a member of the large Src family of non-receptor tyrosine kinases. In GBM, Src and Fyn have been found to be effectors of oncogenic EGFR signaling, which has led to tumor invasion and cancer cell survival. Several studies have implicated Fyn in the regulation of insulin signaling through lipid raft-dependent signaling^32^. Fyn localizes in the lipid raft micro-domains of the plasma membrane where it is associated with lipid raft proteins CD36 that facilitates long-chain fatty acid uptake in skeletal muscle and adipose tissue^33^. In addition, the loss of Fyn improves peripheral tissue insulin sensitivity by relieving a selective negative modulation of AMP kinase activity in adipose tissue and skeletal muscle^34^. However, whether or how Fyn directly regulates tumor cell metabolism remains unknown. Here we show that, upon EGFR activation, Fyn directly phosphorylates 6PGD and enhances 6PGD activity, which consequently activates the PPP.

## Methods

### Materials

Antibodies: mouse monoclonal antibodies against Flag (1:5000, F3165) and anti-Flag M2 affinity gel (A2220) were purchased from Sigma (St. Louis, MO, USA). Rabbit monoclonal antibodies against HA (1:3000, 3724S), β-actin (1:3000, 3700S), phospho-STAT3 Y705 (1:1000, 9145) and phospho-tyrosine (pTyr) (1:2000, 8954S), and rabbit polyclonal antibodies against Fyn (1:3000 for IB, 1:200 for IHC, 4023S) and phospho-EGFR Y1068 (1:1000, 2234) were obtained from Cell Signaling Technology (Danvers, MA, USA). Rabbit polyclonal antibodies against 6PGD (1:3000, 14718-1-AP), GST (1:5000, 10000-0-AP), and Flag (1:5000, 20543-1-AP), and mouse monoclonal antibody against His (1:5000, 66005-1-Ig) were purchased from Proteintech (Wuhan, China). Rabbit polyclonal antibody against phospho-serine (pSer) (1:1000, AB1603) was purchased from Millipore (Darmstadt, Germany). Mouse monoclonal antibody against human IDH1-R132H (1:100, D299-3) was purchased from Medical & Biological Laboratories (Tokyo, Japan). Mouse monoclonal antibody against phospho-threonine (pThr) (1:1000, sc-5267) was purchased from Santa Cruz Biotechnology (CA, USA). The custom-designed rabbit polyclonal antibody against phospho-6PGD Y481 [CTNWTGHGGTVSSSS(pY)NA] (1:1000 for IB, 1:100 for IHC) were obtained from Abclonal Technology (Wuhan, China).

Reagents: puromycin (540411-100MGCN) and hygromycin (400052-20MLCN) were bought from Merck/Millipore (Darmstadt, Germany). DNA transfection reagent PolyJet (100688) was purchased from Signagen Laboratories (Rockville, MD, USA). GelCode Blue Stain Reagent (24590) was obtained from Pierce (Rockford, IL, USA). Recombinant human Fyn (active) (F15-10G-10) and EGFR (active) (E10-11G-10) were purchased from SignalChem (British Columbia, Canada). Recombinant human Src (active) (ab51424) was obtained from Abcam (Cambridge, MA, USA). NADP^+^(N0505), 6-phospho-d-gluconate (P7877), thiamine pyrophosphate (C8754-1G), and sodium arsenate dibasic heptahydrate (A6756-50G) were purchased from Sigma (St. Louis, MO, USA). d-Erythrose 4-phosphate sodium salt (G3117) and Cibacron blue agarose (sc-294028) were from Santa Cruz Biotechnology (CA, USA). ^32^P-labeled adenosine triphosphate (ATP) (NEG002250UC) was purchased from PerkinElmer (Boston, MA, USA). ATP (tlrl-atp) were purchased from InvivoGen (CA, USA).

Kits: NADP/NADPH assay kit (MAK312-1KT) was obtained from Sigma. ROS assay kit (S0033) was obtained from Beyotime (Shanghai, China). CellTiter-Glo ATP quantification kit (G7570) was obtained from Promega.

### Cell culture

U87 (TCHu138), U251 (TCHu58), and HEK293T (GNHu17) cells were obtained from the cell library of the Chinese Academy of Sciences and maintained in high glucose Dulbecco’s modified Eagle’s medium (DMEM) supplemented with 10% fetal bovine serum (FBS). Human primary GSC11 GBM cells were maintained in DMEM/F-12 50/50 supplemented with B27, EGF (10 ng ml^-1^), and bFGF (10 ng ml^-1^)^35^. All cell lines tested negative for mycoplasma.

### Plasmids

PCR-amplified 6PGD was cloned into pCMV-Flag, pCDNA3-HA, pCDH-Flag-Hygromycin, or pCold I-His. PCR-amplified Fyn, Src, or c-Kit was cloned into pCDNA3.0-HA, pCDH-HA-Hygromycin or pCDH-HA-SBP (HB). PCR-amplified EGFR or EGFRvIII was cloned into pCDH-Blastcidin. 6PGD and Fyn mutations were generated using the QuickChange site-directed mutagenesis kit (Stratagene, La Jolla, CA, USA).

The pGIPZ control was generated with the control oligonucleotide 5ʹ-CTCGCTTGGGCGAGAGTAA-3ʹ. pGIPZ 6PGD shRNA was generated with 5ʹ-AGGACTGTCTCCAAAGTTG-3ʹ oligonucleotide targeting the coding region of the 6PGD transcript. pGIPZ Fyn shRNA was generated with 5ʹ-TGAGTTTTCACAAAGGAGA-3ʹ oligonucleotide or 5ʹ-AGACAATGAGTACACAGCA-3ʹ oligonucleotide targeting the coding region of the Fyn transcript.

r6PGD contains same sense mutations of c108a, t109a, c110g, and c111t in the coding region.

### Transfection

Cells were seeded in 35 mm, 60 mm, or 100 mm plates and then transfected with indicated plasmids using PolyJet according to the manufacturer’s instructions.

### Measurement of 6PGD enzymatic activity

6PGD activity was measured at room temperature. 6PGD was immunoprecipitated from the lysates of glioma cells and subjected to 6PGD enzymatic activity assays in the reaction buffer containing 100 mM Tris (PH 7.4), 2 mM 6-phosphogluconate, 10 mM MgCl_2_, 0.1 mM NADP^+^. The change in absorbance at 340 nm owing to increase of NADPH was measured using BioTek Synergy Neo Multi-Mode Plate Reader (BioTek, USA).

### Measurement of 6PGD kinetics

Kinetics of 6PGD was measured at room temperature. His-6PGD WT and His-6PGD Y481F were purified from bacteria and subjected to kinetics measurements in the reaction buffer containing 100 mM Tris (PH 7.4), 2 mM 6-PG, 10 mM MgCl_2_. The final concentration of NADP^+^ was from 2/15 to 1/300 mM. The velocity was measured using BioTek Synergy Neo Multi-Mod Plate Reader (BioTek, USA). The *K*_m_ and V_max_ was calculated by nonlinear regression/Michaelis–Menten using GraphPad Prism software. The *k*_cat_ was calculated using V_max_/(6PGD enzyme concentration).

### Mass spectrometry analysis of PPP flux

High performance liquid chromatography-mass spectrometry (HPLC-MS) grade acetonitrile and methanol are the product of Fisher Scientific. d-Glucose-1,2-^13^C_2_ and d-( + )-glucose were purchased from Sigma (St. Louis, MO, USA). Fructose-6-phosphate (F-6-P), G-6-P, R-5-P, and Ru-5-P were the products of Santa Cruz Biotechnology (Dallas, Texas, USA). Sedoheptulose-7-phosphate (S-7-P) was purchased from Cayman Chemical (Ann Arbor, Michigan, USA). Ammonium acetate and ammonium hydroxide were of liquid chromatography mass spectrometry (LC-MS) grade and purchased from Anpel Laboratory Technologies Inc (Shanghai, China).

The cell mixture was added 80% volume of methanol and processed by five cycles of 1-min ultra-sonication and 1-min interval in ice-water bath. After centrifugation at 16,000 *g* and 4 °C for 15 min, 800 μl of supernatant was evaporated to dryness under nitrogen gas. The residue was reconstituted in 100 μl of 50% aqueous acetonitrile, and the supernatant was performed in UPLC-MS/MS analysis.

Ultra performance liquid chromatography-mass spectrometry/mass spectrometry (UPLC-MS/MS) analysis was performed on a Waters Acquity UPLC system (Waters, Milford, MA) coupled to a Triple Quad™ 5500 tandem mass spectrometer (AB Sciex, Framingham, MA). The samples were separated using a Waters UPLC BEH Amide column (100 mm × 2.1 mm, 1.7 μm) with a linear gradient elution system of mobile-phase (A) 10 mM ammonium acetate and 0.1% ammonium hydroxide in water and (B) 10 mM ammonium acetate and 0.1% ammonium hydroxide in acetonitrile/water (90/10, v/v). The gradient elution conditions were 0–1 min, 95% B; 2 min, 70% B; 6 min, 60% B; 6.1 min, 50% B; 7 min, 50% B; 7.1 min, 95% B; 12 min, 95% B. The column temperature was 30 °C. The injection volume was 4 μl. The flow rate was maintained at 250 μl min^-1^.

The analytes separated from column were ionized in an electro spray ionization source in negative mode (ESI–). Source temperature: 500 °C, curtain gas (CUR): 25 psi, ion source gas 1 (GAS1): 50 psi, ion source gas 2 (GAS2): 50 psi, collision gas (CAD): 8 psi, ion spray voltage (IS): –4500 V, entrance potential (EP): –10 V, collision cell exit potential (CXP1): –10 V. The dwelling time was set at 20 ms. The multiple reaction monitoring (MRM) was used to acquire data in optimized MRM transition (precursor > product), declustering potential (DP), and collision energy (CE) as Supplementary Table 1. AB Sciex Analyst software (version 1.5.2) was used to control instruments and acquire data.

Analyst (version 1.6.3, AB Sciex) was used to analyze data. Using the default parameters and assisting manual inspection to ensure the qualitative and quantitative accuracy of each compound, extract and output chromatographic retention time and peak area.

### Mass spectrometry analysis of 6PGD phosphorylation

Immunoprecipitated Flag-6PGD using anti-Flag antibody from U87/EGFR cells treated with or without EGF (100 ng ml^-1^) for 30 min. The precipitated complexes were boiled at 95 ℃ for 10 min. Flag-6PGD were separated from the complexes using sodium dodecyl sulfate–polyacrylamide gel electrophoresis (SDS-PAGE) gel and trypsinized^36^. In brief, peptides digested with a modified in-gel trypsin procedure were extracted by removing the ammonium bicarbonate solution, followed by washes with a solution containing 50% (v/v) acetonitrile and 5% (v/v) formic acid. The samples were then dried and reconstituted in 5 μl of solvent A [5% (v/v) acetonitrile, 0.005% hepta-fluorobutyric acid, 0.4% acetic acid]. The peptides were analyzed by LC-MS/MS on a Q Exactive mass spectrometer (Thermo Fisher Scientific, Waltham, MA).

### In vitro kinase assay

In brief, bacterially purified recombinant His-6PGD (2 μg) was incubated with HEK293T-purified WT or KD HB-Fyn (0.5 μg) or commercial GST-Fyn, GST-Src, or GST-EGFR (active, 0.5 μg) in kinase buffer (60 mM HEPES pH 7.5, 5 mM MgCl_2_, 5 mM MnCl_2_, 3 µM Na_3_VO_4_ and 1.25 mM DTT). In al, 5 μCi ^32^P-labeled ATP or 20 µM ATP was added in the kinase buffer to start the reaction. The reactions were performed in a total volume of 50 μl at 37 °C for 30 min and then terminated by adding SDS-PAGE loading buffer.

### Intracellular ROS production

Intracellular ROS levels were measured using a ROS measuring kit. The amount of intracellular ROS was measured by detecting dichlorodihydrofluorescein, which is the cleavage product of carboxy-H_2_DCFDA by ROS. In all, 1 × 10^6^ cells were seeded in six-well plate. Twenty-four hours after seeding, cells were washed with phosphate-buffered saline (PBS) and loaded with 10 mM carboxy-H_2_DCFDA for 30 min. The cells were harvested, resuspended in PBS and analyzed using fluorescence-activated cell sorter (FACS).

### Intracellular Ru-5-P measurement

To determine cellular concentration of Ru-5-P, the cell lysates were centrifuged in a cold room at 4 °C for 10 min at maximum speed, and the supernatants were applied to Amicon Ultra tubes with 5 kDa cut off filter (Millipore). The final reaction buffer (100 μl) contains: 50 mM imidazole pH = 7.6, 10 mM NAD^+^, 6 mM MgCl_2_, 5 mM sodium arsenate, 0.08 mM d-Erythrose 4-phosphate and 0.01% thiamine pyrophosphate, 2 μg glyceraldehyde-3-phosphate dehydrogenase, 1 μg transketolase. The reaction was carried out at room temperature for 30 min. Then 2 μg ribulose-phosphate 3-epimerase was added and the increase absorption of OD_340_ was measured.

### Intracellular ATP measurement

Intracellular ATP concentration was measured using a CellTiter-Glo ATP quantification kit according to the instruction of manufacturer.

### Cibacron blue agarose pulldown assay

The NADP^+^ binding ability of 6PGD was determined by the affinity of 6PGD to Cibacron blue agarose, which mimics NADP^+^. U87/EGFR cells transfected with Flag-6PGD in 60 mm dish were harvested and incubated with 10 μl Cibacron blue agarose at 4 °C for 30 min. After washing with cell lysis buffer for three times, Cibacron blue agarose was boiled and subjected to western blotting.

### Protein expression, purification, and verification

pCold I-His-6PGD WT or Y481F was transformed into BL21-CodonPlus competent cells. His-6PGD proteins were extracted by sonication of these BL21 cells, which were subjected to isopropyl β-D-1-thiogalactopyranoside (IPTG) (0.5 mM) induction for 24 h at 15 ℃, followed by the purification using HisTrip HP column by The AKTA FPLC System (GE Healthcare). The column was washed using wash buffer (containing 50 mM Tris-HCl, pH = 8.0; 300 mM NaCl; 20 mM imidazole) until the absorbance reached the baseline. The proteins were eluted by an imidazole gradient elution buffer (containing 50 mM Tris-HCl, pH = 8.0; 300 mM NaCl; 0-250 mM imidazole). The proteins were further purified using Superdex 200 Increase 10/300 GL column by the AKTA FPLC System. The purified proteins were concentrated using Amicon® Ultra-4 centrifugal filter concentrator (Millipore) and quantitated by BCA protein assay kit (Pierce).

HEK293T/EGFR cells were transfected with pCMV-Flag-6PGD WT or Y481F. Flag-6PGD proteins were immunoprecipitated from these cells by anti-Flag M2 affinity gel (Sigma). The immunoprecipitated complex were washed three times using hash wash buffer containing 0.1% SDS followed by elution with Flag peptides. The Flag peptides were removed and proteins were concentrated using Amicon® Ultra-0.5 centrifugal filter concentrator (Millipore) and quantitated by BCA protein assay kit (Pierce).

In all, 1 μg His-6PGD WT and Y481F, or 2 μg Flag-6PGD WT and Y481F proteins were subjected to SDS-PAGE. The protein purity was verified by GelCode Coomassie blue stain reagent (Pierce) following the instructions of the manufacturer and western blotting. For Coomassie blue staining, the gel was washed with deionized water for 15 min and then incubated with GelCode stain reagent for 1 h. The reagent was next replaced with ultrapure water for 1 h. The gel image was obtained by gel imaging systems (Tanon).

### ITC analysis

In vitro kinase assays were performed by incubating bacterial purified recombinant His-6PGD WT or Y481F with or without recombinant active Fyn. After the reaction, His-6PGD variants were pulldown using nickel NTA agarose beads. The beads were washed using wash buffer containing 20 mM imidazole for three times, and the proteins were eluted by an elution buffer containing 250 mM imidazole. Amicon® Ultra-0.5 centrifugal filter concentrator (Millipore) were used to concentrate and desalt the proteins.

ITC assays were performed by the ITC200 Micro-calorimeter (MicroCal). His peptides, Flag peptides, purified His-6PGD variants, or purified Flag-6PGD variants were diluted to a final concentration of 50 μM (containing 50 mM Tris-HCl, pH 7.4; 20 mM KCl; and 10 mM MgCl_2_). His and Flag peptides were used as the negative control. NADP^+^ was diluted to a final concentration of 1 mM (containing 50 mM Tris-HCl, pH 7.4; 20 mM KCl, and 10 mM MgCl_2_). Temperature equilibration was allowed for 1–2 h till to 25 °C prior to the experiment. The calorimetric cell was washed three times using dialysis buffer (containing 50 mM Tris-HCl, pH 7.4; 20 mM KCl, and 10 mM MgCl_2_). All solutions were thoroughly degassed by centrifugation before use.

His peptides, Flag peptides, or 6PGD variants were slowly injected into the calorimetric cell to avoid air bubbles. The experiment was conducted by consecutively injecting NADP^+^ solution into the calorimetric cell containing peptides or 6PGD variants. In all experiments, the initial injection of 0.5 μl of NADP^+^ solution was discarded to eliminate the effect of titrant diffusion across the syringe tip during the equilibration process, and each dataset consisted of 20 injections of 2 μl each of 1 mM NADP^+^ into the calorimetric cell containing 250 μl of 50 μM peptides or 6PGD variants. The heat of dilution was negligible in all cases. The binding constant and other thermodynamic parameters were determined by fitting the integrated titration data using the single binding site model by the nonlinear least-squares method implemented in MicroCal Origin software (version 7.0). The titration enthalpy data were corrected for the small heat changes in titrations of small molecular solution into the dialysis buffer.

### Cross-linking assay

U87/EGFR cells transfected with Flag-6PGD in 35 mm dish was harvested using 200 μl cell lysis buffer. Glutaraldehyde was added in 50 μl cell lysates and mixed to a final concentration of 0.025%. The cross-linking assay was carried out in room temperature for 15 min, and then 10 μl 1 M Tris-HCl (pH = 7.5) was added to stop the reaction.

### BrdU incorporation assay

Cells were plated on glasses for 24 h and were treated with BrdU (20 μM) for 20 min. Then cells were washed and fixed in 4% paraformaldehyde (PFA) for immunofluorescence assays.

### Cell proliferation assay

In all, 5 × 10^4^ cells in DMEM with 10% fetal calf serum were seeded in six-well plates. Cells were counted with Thermo countess II FL.

### Colony formation assay

For colony formation assays, 5000 cells per well were plated in six-well plate in triplicates and cultured for 14 days before staining viable colonies with nitro blue tetrazolium (Sigma).

### Cell death assay

In total, 1 × 10^6^ cells were seeded in six-well plates. Twenty-four hours after X-ray radiation (10 Gy) treatment, cells were harvested with trypsin using 600 *g* centrifugation. Cells was stained with Trypan blue and counted using Thermo countess II FL.

### NADPH/NADP^+^ ratio

NADPH/NADP^+^ ratios were determined by measuring NADPH/NADP^+^ concentrations according to the protocol of NADP/NADPH assay (Sigma). In brief, NADP^+^/NADPH assay is based on a glucose dehydrogenase cycling reaction, in which the formed NADPH reduces a probe into a highly fluorescent product. The fluorescence intensity of this product, measured at ex = 530 nm/em = 585 nm.

### Intracranial injection, bioluminescence imaging, and hematoxylin and eosin (H&E) staining

In Figs. 4g and 4h, approximately 2 × 10^5^ (in 5 μl of DMEM per mouse) luciferase-expressing 6PGD-depleted U87/EGFRvIII cells with reconstituted expression of 6PGD WT or Y481F were intracranially injected into randomized 8-week-old female athymic nude mice. Briefly, a small hand-controlled twist drill that is 1 mm in diameter is used to make a hole in the animal’s skull. The cell suspension is drawn up into the cuffed Hamilton syringe. The needle of the Hamilton syringe is slowly lowered into the central hole of the guide screw until the cuff rests on the screw surface. The cell suspension is slowly injected into the mouse’s brain^37^. Five mice per group in each experiment were included.

Seven, 9, or 11 days after inoculation, the mice were intraperitoneally injected with 100 μl of 7.5 mg ml^-1^ d-luciferin (Xenogen) and subsequently anesthetized with isoflurane inhalation. Bioluminescence imaging with a CCD camera (IVIS, Xenogen) was initiated 10 min after injection. Bioluminescence from the region of interest was defined manually. Background was defined using a region of interest from a mouse that was not given an intraperitoneal injection of d-luciferin. All bioluminescent data were collected and analyzed using IVIS software.

Eleven days after inoculation and bioluminescence imaging, animals were sacrificed, and the brain of each mouse was harvested, fixed in 4% formaldehyde, and embedded in paraffin. Tumor formation and phenotype were determined by histological analysis of H&E-stained sections. Calculation to formulate tumor volume was V = ab^2^/2. Data represent the mean ± SD of five mice.

In Figs. 5f and 5g, approximately 1 × 10^5^ (in 5 μl of DMEM per mouse) luciferase-expressing 6PGD-depleted U87/EGFRvIII cells with reconstituted expression of 6PGD WT or Y481F were intracranially injected into randomized 8-week-old female athymic nude mice and then subjected to IR (γ) radiation (4 Gy) in 5 and 10 days after inoculation. Five mice in each group were included.

Four, 9, 12, 15, 17, 19 days after inoculation, bioluminescence imaging of mice was carried out as described above. Survival durations of the implanted mice were compared.

The use of mice was in compliance with ethical regulations and was approved by the institutional review board at Institute of Biochemistry and Cell Biology.

### IHC analysis

The tissue sections from paraffin-embedded human GBM and astrocytoma specimens were stained with antibodies as indicated. We quantitatively scored the tissue sections according to the percentage of positive cells and staining intensity. We rated the intensity of staining on a scale of 0–3: 0, negative; 1, weak; 2, moderate; and 3, strong. We assigned the following proportion scores: X means X% of the tumor cells were stained (0 ≤ X ≤ 100). The score (H-score) was obtained by the formula: 3 × percentage of strongly staining area + 2 × percentage of moderately staining area + 1 × percentage of weakly staining area, giving a range of 0–300. Scores were compared with overall survival, defined as the time from the date of diagnosis to death or last known date of follow-up. All patients received standard adjuvant radiotherapy after surgery, followed by treatment with an alkylating agent (TMZ in most cases). The use of human brain tumor specimens and the database was approved by the Institutional Review Board at XinHua Hospital School of Medicine and the institutional review board at First Affiliated Hospital of Wenzhou Medical College. Informed consent was obtained from all patients.

### Statistical analysis

We determined the significance of differences in the human glioma data using Pearson’s correlation test and Student’s *t*-test (two tailed). *p* < 0.05 was considered to be significant.

### Reporting summary

Further information on experimental design is available in the Nature Research Reporting Summary linked to this article.

## Supplementary information

Supplementary Information

Source Data

Reporting Summary

## Data Availability

All data supporting the conclusions are available from the authors on reasonable request. A reporting summary for this article is available as a Supplementary Information file. The source data underlying Figs 1a, 1c, d, 1h, 2a-g, 3a-d, 4a-k, 5a-d, 5f-g, and 6b-d, and Supplementary Figs. 1, b, c, 1f, 1h, 2a-h, 3a-e, 4a-g, 5a-d, and 6e-f are provided as a Source Data file.
