# Supplementary material for: Tyrosine phosphorylation activates 6-phosphogluconate dehydrogenase and promotes tumor growth and radiation resistance
Source: Nat Commun. 2019 Mar 1;10:991. doi: 10.1038/s41467-019-08921-8 (PMC6397164; doi:10.1038/s41467-019-08921-8)
Supplement: Supplementary file 1 — Supplementary Information [file 41467_2019_8921_MOESM1_ESM.pdf]

Supplementary Information

Tyrosine phosphorylation activates 6-phosphogluconate dehydrogenase and promotes tumor growth and radiation resistance

Liu et al.

Supplementary Fig. 1

a

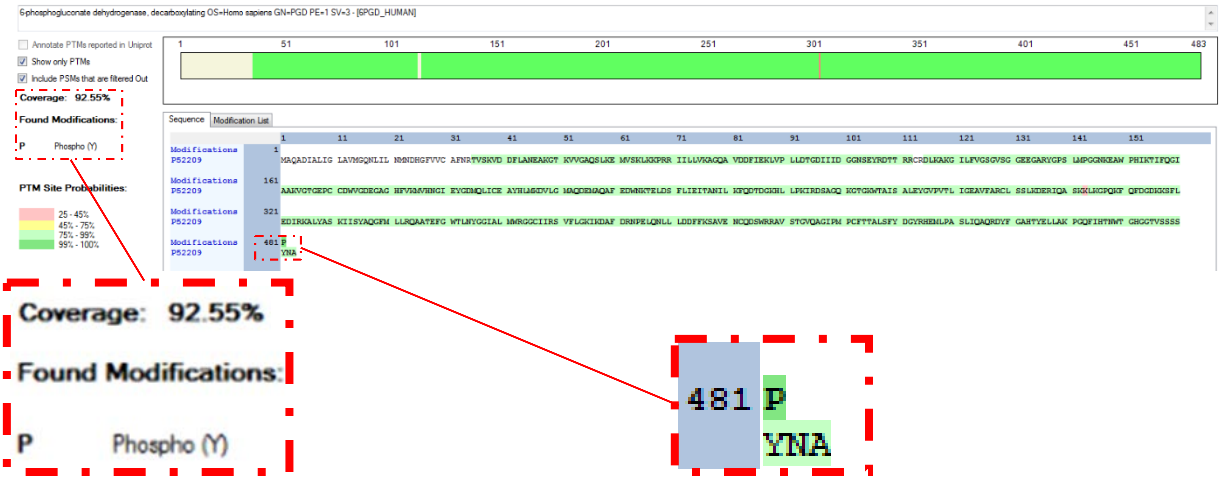

	b^{3+}	b^{4+}	Seq.
33	1183. 22704	887. 67210	S
34	1264. 23692	948. 42951	Y-Phospho

Charge: +4,
Monoisotopic m/z: 999.20270 Da (+0.54 mmu/+0.54 ppm),
RT: 73.1367 min

b **c** **d** **Shown in next page.**

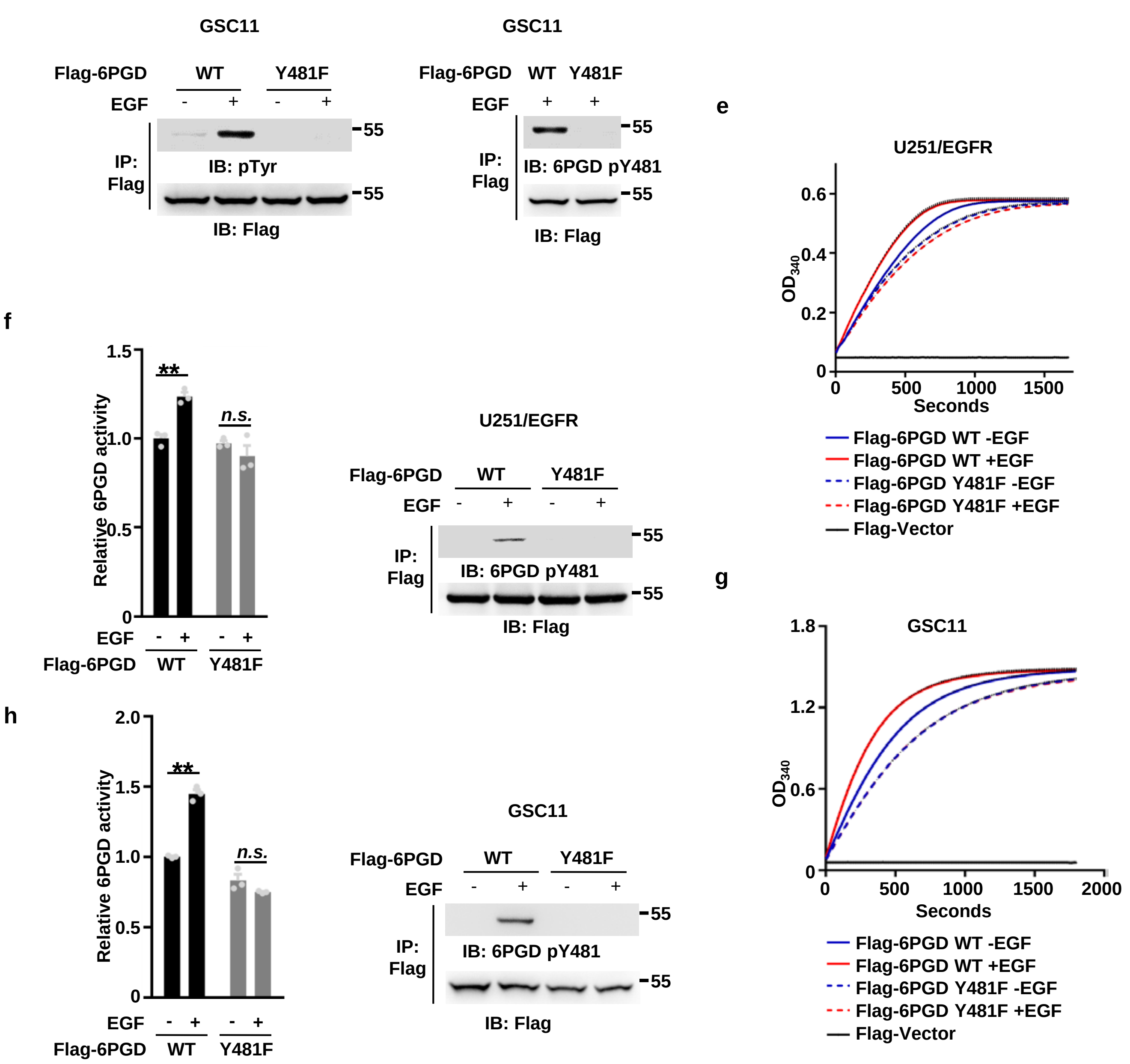

Supplementary Fig.1 (continued)

d

```
CLUSTAL O(1.2.4) multiple sequence alignment


Human                           --MAQADIALIGLAVMGQNLILNMNDHGFVVCAFNRTVSKVDDFLA---NEAKGTKVVGA    55
Rat                             --MAQADIALIGLAVMGQNLILNMNDHGFVVCAFNRTVSKVDDFLA---KEAKGTKVIGA    55
Mouse                           --MAQADIALIGLAVMGQNLILNMNDHGFVVCAFNRTVSKVDDFLA---NEAKGTKVVGA    55
Sheep                           --MAQADIALIGLAVMGQNLILNMNDHGFVVCAFNRTVSKVDDFLA---NEAKGTKVLGA    55
Bovine                          --MAQADIALIGLAVMGQNLILNMNDHGFVVCAFNRTVSKVDDFLA---NEAKGTKVLGA    55
Dog                             --MAEADVALIGLAVMGQNLILNMNDHGFVVCAFNRTVSKVDDFLA---NEAKGTKVVGA    55
Pig                             --MAQADIALIGLAVMGQNLILNMNDHGFVVCAFNRTVSKVDDFLA---NEAKGTRVVGA    55
Giant_panda                     ---SRADIALIGLAVMGQNLILNMNDHGFVVCAFNRTVSKVDDFLA---NEAKGTKVVGA    54
Horse                           ----RADIALIGLAVMGQNLILNMNDHGFVVCAFNRTVSKVDDFLA---NEAKGTKVVGA    53
Atlantic_salmon                 --MAEADIALIGLAVMGQNIILNMNDHGFVVCAYNRTVSKVHDFLA---NEAKGTKIIGA    55
Osmerus_mordax                  --MAQADIALIGLAVMGQNIILNMNDNGFVVCAYNRTVSKVHDFLK---NEAKGTKVIGA    55
Black_porgy                     --MAEADIALIGLAVMGQNLIMNMNDHGFVVCAYNRTVSKVHDFLK---NEAKGSKVVGA    55
Anthurium_amnicola              MSKPVGDFGLIGLAVMGQNLILNINDHGYTVVAYNRTVSKVDHFLA---NEAKGTNIVGA    57
Chlamydomonas_reinhardtii       --MAVAEIGLVGLAVMGQNLALNIAEKGFPISVYNRSYDKTEAAVKRAQKEGLGEKLHGY    58
Zebrafish                       --MAQADIALIGLAVMGQNLILNMNDHGFVVCAFNRTVSKVHDFLN---NEAKGTKVIGA    55
Caenorhabditis_elegans          --MAEADIAVIGLAVMGQNLILNMNDHGFTVCAFNRTVKLVDDFLA---NEAKGTKIIGA    55
Drosophila_melanogaster         -MSGQADIALIGLAVMGQNLILNMDEKGFVVCAYNRTVAKVKEFLA---NEAKDTKVIGA    56
Yeast                           MSKAVGDLGLVGLAVMGQNLILNAADHGFTVVAYNRTQSKVDRFLA---NEAKGKSIIGA    57
                                     .:..::********: :*  ::*: : .:**:   ..  :    :*. .  : *

Human                           QSLKEMVSKLKKPRRIILLVKAGQAVDDFIEKLVPLLDTGDIIIDGGNSEYRDTTRRCRD    115
Rat                             KSLKDMVSKLKKPRRVILLVKAGQAVDDFIEKLVPLLDTGDIIIDGGNSEYRDTTRRCQD    115
Mouse                           QSLKDMVSKLKKPRRVILLVKAGQAVDDFIEKLVPLLDTGDIIIDGGNSEYRDTTRRCRD    115
Sheep                           HSLEEMVSKLKKPRRIILLVKAGQAVDNFIEKLVPLLDIGDIIIDGGNSEYRDTMRRCRD    115
Bovine                          HSLEEMASKLKKPRRIILLVKAGQAVDDFIEKLVPLLDIGDIIIDGGNSEYRDTMRRCRD    115
Dog                             HSLEEMVSKLKKPRRIILLVKAGQAVDDFIGKLVPLLNTGDIIIDGGNSEYRDTTRRCRD    115
Pig                             RSLEEMVSMLKKPRRIILLVKAGQAVDDFIEKLVPLLDTGDIIIDGGNSEYRDTTRRCRD    115
Giant_panda                     HSLEEMVSKLKTPRRIILLVKAGQAVDDFIDRLVPLLDTGDIIIDGGNSEYRDTTRRCRS    114
Horse                           HSLEEMVSKLKKPRRIILLVKAGQAVDDFIEKLVPLLDTGDIIIDGGNSEYRDTTRRCRD    113
Atlantic_salmon                 ESLEDMVAKLKKPRRIVMLVKAGQAVDDFIDKLVPLLEAGDIIIDGGNSEYRDTTRRCKS    115
Osmerus_mordax                  ESLEDMVSKLKKPRRIILLVKAGQAVDDFIDKLVPLLEAGDIIIDGGNSEYRDTTRRCQS    115
Black_porgy                     ESLEDMVSKLKKPRRIILLVKAGQAVDDFIDKLVPLLEAGDIIIDGGNSEYRDTTRRCQS    115
Anthurium_amnicola              HSIEDLAKKLKTPRKIMLLVKAGSAVDDFIEQIIPHLEKGDIIIDGGNSHFPDTIRRCKY    117
Chlamydomonas_reinhardtii       EQVKDFVQSLQRPRRVIILVKAGAPVDQTIDQLCEFMEPGDIIIDGGNEWYENTEKRMAK    118
Zebrafish                       ESLEDMVSKLKKPRRIILLVKAGQAVDDFIDKLVPLLEPGDIIIDGGNSEYRDTTRRCKS    115
Caenorhabditis_elegans          HSIEEMCKKLKRPRRVMMLIKAGTPVDMMIDAIVPHLEEGDIIIDGGNSEYTDSNRRSEQ    115
Drosophila_melanogaster         DSLEDMVSKLKSPRKVMLLVKAGSAVDDFIQQLVPLLSAGDVIIDGGNSEYQDTSRRCDE    116
Yeast                           TSIEDLVAKLKKPRKIMLLIKAGAPVDTLIKELVPHLDKGDIIIDGGNSHFPDTNRRYEE    117
                                 .::::   *: **::::*:***  **  *  :   :. **:******. : :: :*

Human                           LKAKGILFVGSGVSGGEEGARYGPSLMPGGNKEAWPHIKTIFQGIAAKVGTGEPCCDWV-    174
Rat                             LKAKGILFVGSGVSGGEEGARYGPSLMPGGNKEAWPHIKTIFQAIAAKVGTGEPCCDWV-    174
Mouse                           LKAKGILFVGSGVSGGEEGARYGPSLMPGGNKEAWPHIKAIFQAIAAKVGTGEPCCDWV-    174
Sheep                           LKDKGILFVGSGVSGGEDGARYGPSLMPGGNKEAWPHIKAIFQGIAAKVGTGEPCCDWV-    174
Bovine                          LKEKGILFVGSGVSGGEDGARYGPSLMPGGNKEAWPHIKAIFQGIAAKVGTGEPCCDWV-    174
Dog                             LKAKGILFVGSGVSGGEEGARYGPSLMPGGDKEAWPHIKTIFQGIAAKVGSGEPCCDWV-    174
Pig                             LKAKGILFVGSGVSGGEEGARYGPSLMPGGNREAWPHLKEIFQSIAAKVGTGEPCCDWV-    174
Giant_panda                     LKAKGILFVGSGVSGGEEGARYGPSLMPGGDKEAWPHIKTIFQGIAAKVGTGEPCCDWV-    173
Horse                           LKAKGILFVGSGVSGGEEGARHGPSLMPGGNKEAWPHIKTIFQGIAAKVGTGEPCCDWAK    173
Atlantic_salmon                 LKEKNLLFVGSGVSGGEEGARYGPSLMPGGHKEAWPHIKEIFQSIAAKVGTGEPCCDWV-    174
Osmerus_mordax                  MKDKKLLFVGSGVSGGEEGARYGPSLMPGGHKEAWPHIKDIFQSIAAKVGTGEACCDWV-    174
Black_porgy                     LKAKGLLFVGSGVSGGEEGARYGPSLMPGGHIEAWPHIKDIFQSIAAKVGTGEPCCDWV-    174
Anthurium_amnicola              LEEKGFLFIGCGVSGGEDGARYGPSLMPGGSKAAWEHVKPIFQSISAK-VKDEPCCEWV-    175
Chlamydomonas_reinhardtii       VAAKGILYMGMGVSGGEEGARRGPSMMPGGSPEAYSHIKSVVEKVAAQ-VDDGPCVMYI-    176
Zebrafish                       LKEKNLLFVGSGVSGGEDGARYGPSLMPGGHKDAWPHLKDIFQSIAAKVGTGEPCCDWV-    174
Caenorhabditis_elegans          LAAKGIMFVGCGVSGGEEGARFGPSLMPGGNPKAWPHLKDIFQKIAAK-SNGEPCCDWV-    173
Drosophila_melanogaster         LAKLGLLFVGSGVSGGEEGARHGPSLMPGGHEAAWPLIQPIFQAICAK-ADGEPCCEWV-    174
Yeast                           LTKQGILFVGSGVSGGEDGARFGPSLMPGGSAEAWPHIKNIFQSIAAK-SNGEPCCEWV-    175
                                :    ::::* ******:*** ***:****   *:  :: :.: :.*:   .  *  :
```

Supplementary Fig.1 (continued)

d (continued)

```
Human                       -GDEGAGHFVKMVHNGIEYGDMQLICEAYHLMKDVLGMAQDEMAQAFEDWNKTELDSFLI   233
Rat                         -GDEGAGHFVKMVHNGIEYGDMQLICEAYHLMKDVLGMRHEEMAQAFEDWNKTELDSFLI   233
Mouse                       -GDEGAGHFVKMVHNGIEYGDMQLICEAYHLMKDVLGMRHEEMAQAFEEWNKTELDSFLI   233
Sheep                       -GDDGAGHFVKMVHNGIEYGDMQLICEAYHLMKDVLGLGHKEMAKAFEEWNKTELDSFLI   233
Bovine                      -GDEGAGHFVKMVHNGIEYGDMQLICEAYHLMRDVLCMGHAEMAKAFEEWNKTELDSFLI   233
Dog                         -GDEGAGHFVKMVHNGIEYGDMQLICEAYHLMRDVLGMEHNGMAEAFEEWNKTELDSFLI   233
Pig                         -GDEGAGHFVKMVHNGIEYGDMQLICEAYHLMKDVLGMEQHEMAKAFEEWNKTELDSFLI   233
Giant_panda                 -GDEGAGHFVKMVHNGIEYGDMQLICEAYHLMRDVLGMGHDEMAKVFEEWNKTELDSFLI   232
Horse                       VGSEGAGHFVKMVHNGIEYGDMQLICEAYHLMKDVLGMEHNEMAKAFEEWNKTELDSFLI   233
Atlantic_salmon             -GDEGAGHFVKMVHNGIEYGDMQLICEAYHLMKDVLGMDHDEMAQAFEDWNKTELDSFLI   233
Osmerus_mordax              -GDEGAGHFVKMVHNGIEYGDMQLICEAYHLMKDVLGMDHDEMAKAFNEWNKTELDSFLI   233
Black_porgy                 -GDEGAGHFVKMVHNGIEYGDMQLICEAYHLMKDVLGMDHDEMAQAFDKWNKTELDSFLI   233
Anthurium_amnicola          -GETGAGHYVKMVHNGIEYGDMQLICEAYHLMKEGLDLTHDEIGDVFESWNKEELDSFLI   234
Chlamydomonas_reinhardtii   -GGGGAGNFVKMVHNGIEYGDMQLISEAYDVLKTLGGLNNEELAAVFKEWNDAELKSFLV   235
Zebrafish                   -GDEGAGHFVKMVHNGIEYGDMQLICEAYHLMKDVLCMNHDEMAQVFEQWNKTELDSFLI   233
Caenorhabditis_elegans      -GNAGSGHFVKMVHNGIEYGDMQLIAEAYHLLSKAVELNHDQMAEVLDDWNKGELESFLI   232
Drosophila_melanogaster     -GDGGAGHFVKMVHNGIEYGDMQLICEAYHIMKS-LGLSADQMADEFGKWNSAELDSFLI   232
Yeast                       -GPAGSGHYVKMVHNGIEYGDMQLICEAYDIMKRIGRFTDKEISEVFDKWNTGVLDSFLI   234
                             *  *:*::****************.***.::     :    :.  : .**   *.***:

Human                       EITANILKFQDT-DGKHLLPKIRDSAGQKGTGKWTAISALEYGVPVTLIGEAVFARCLSS   292
Rat                         EITANILKFQDT-DGKELLPKIRDSAGQKGTGKWTAISALEYGMPVTLIGEAVFARCLSS   292
Mouse                       EITANILKYRDT-DGKELLPKIRDSAGQKGTGKWTAISALEYGMPVTLIGEAVFARCLSS   292
Sheep                       EITASILKFQDA-DGKHLLPKIRDSAGQKGTGKWTAISALEYGVPVTLIGEAVFARCLSS   292
Bovine                      EITANILKFQDA-DGKHLLPKIRDSAGQKGTGKWTAISALEYGVPVTLIGEAVFARCLSS   292
Dog                         EITANILKFRDS-DGQHLLPKIRDSAGQKGTGKWTAISALEYGVPVTLIGEAVFARCLSS   292
Pig                         EITANILKFQDA-DGKHLLPKIRDSAGQKGTGKWTAISALEYGVPVTLIGEAVFARCLSS   292
Giant_panda                 EITANILKFQDS-DGKHLLPKIRDSAGQKGTGKWTAISALEYGVPVTLIGEAVFARCLSS   291
Horse                       EITANILKFQDT-DGKYLLPKIRDSAGQKGTGKWTAISALEYGVPVTLIGEAVFARCLSS   292
Atlantic_salmon             EITAGILKFRDT-DGTHLLPKIRDAAGQKGTGKWTAISALEYGTPVTLIGEAVFARCLSA   292
Osmerus_mordax              EITANILKFRDA-DGTHLLPKIRDSAGQKGTGKWTAISALEYGTPVTLIGEAVFARCLSS   292
Black_porgy                 EITANIFKYRDS-DGTHLLPKIRDSAGQKGTGKWTAISALEYGTPVTLIGEAVFARCLSS   292
Anthurium_amnicola          EITKDIMRFKDE-DGKPLVEKIKDTAGQKGTGKWTAISSLDLGVPVTLIGEAVYSRTLSS   293
Chlamydomonas_reinhardtii   EITAIIVNKKDDQAPGYLVDQIVDQTGSKGTGKWTVQQAAELAVAAPTMASALDARYMSA   295
Zebrafish                   EITANILKFKDA-DGTNLLPKIRDSAGQKGTGKWTAISALEYGTPVTLIGEAVFARCLSS   292
Caenorhabditis_elegans      EITANILKYRDE-QGEPIVPKIRDSAGQKGTGKWTCFAALEYGLPVTLIGEAVFARCLSA   291
Drosophila_melanogaster     EITRDILKYKDG-KG-YLLERIRDTAGQKGTGKWTAIAALQYGVPVTLIGEAVFSRCLSA   290
Yeast                       EITRDILKFDDV-DGKPLVEKIMDTAGQKGTGKWTAINALDLGMPVTLIGEAVFARCLSA   293
                            ***  *..  *      :: :* * :*.*******   : : .  .  :..*: :* :*:

Human                       LKDERIQASKKLKGPQKF--QFDGDKKSFLEDIRKALYASKIISYAQGFMLLRQAATEFG   350
Rat                         LKEERVQASRKLKGPKMV--QLEGSKQAFLEDVRKALYASKIISYAQGFMLLRQAATEFG   350
Mouse                       LKEERVQASQKLKGPKVV--QLEGSKKSFLEDIRKALYASKIISYAQGFMLLRQAATEFG   350
Sheep                       LKDERIQASKKLKGPQNI--PFEGDKKSFLEDIRKALYASKIISYAQGFMLLRQAATEFG   350
Bovine                      LKDERIQASKKLKGPQNV--PFEGDKKSFLEDIRKALYASKIISYAQGFMLLRQAATEFG   350
Dog                         LKDERIQASKKLKGPQKM--QFEGDKKAFLEDIRKALYASKIISYAQGFMLLRQAATEFG   350
Pig                         LKDERVQASKKLKGPQKI--QFSGDKKSFLEDIRKALYASKIISYTQGFMLLRQAAAEFG   350
Giant_panda                 LKDERIQASKKLKGPQKI--QFEGDKKAFLEDIRKALYASKIVSYAQGFMLLRQAATELG   349
Horse                       LKDERIQASGKLKGPRKI--QFEGDKKSFLEDIRKALYASKIISYAQGFMLLRQAATEFG   350
Atlantic_salmon             LKDERVEASRSLAGPQGA--KFNGDKASFLEDIRKALYASKIISYAQGFMLLRQAAKEFG   350
Osmerus_mordax              LKEERVEASKSLSGPEGT--KFSGNKTSFLEDIRKALYASKIISYAQGFMLLRQAAKEFG   350
Black_porgy                 LKDERVEASRSLSGPQGV--KFSGDKEAFLEDIRKALYASKIISYAQGFMLLRQAAKEFG   350
Anthurium_amnicola          IKDERVRASKILPGPKNK-K-YSGDKKLFVKQLGQALYASKLISYAQGFMLMREAAKDNN   351
Chlamydomonas_reinhardtii   LKSERVAASKSFTSCVTPGPVAGVDKAQLINDVRAALYASKICSYAQGMNIIKAKSVEMK   355
Zebrafish                   LKDERVQASKSLSGPQGV--KFTGNKEQFLEDIRKALYASKIISYAQGFMLLRQAALEFG   350
Caenorhabditis_elegans      LKDERVRASKQLPRPQVSPDTVVQDKRVFIKQISKALYASKIVSYAQGFMLLAEASKQFN   351
Drosophila_melanogaster     LKDERVQASSVLKGPSTK--AQVANLTKFLDDIKHALYCAKIVSYAQGFMLMREAARENK   348
Yeast                       IKDERKRASKLLAGPTVP-KDAIHDREQFVYDLEQALYASKIISYAQGFMLIREAARSYG   352
                            :*.**  **  :            .   :: ::  ***.:*: **:**: ::   : .
```

Supplementary Fig.1 (continued)

d (continued)

```
Human                           WTLNYGGIALMWRGGCIIRSVFLGKIKDAFDRNPELQNLLLDDFFKSAVENCQDSWRRAV     410
Rat                             WTLNYGGIALMWRGGCIIRSVFLGKIKDAFERNPELQNLLLDDFFKSAVDDCQDSWRRVI     410
Mouse                           WTLNYGGIALMWRGGCIIRSVFLGKIKDAFERNPELQNLLLDDFFKSAVDNCQDSWRRVI     410
Sheep                           WTLNYGGIALMWRGGCIIRSVFLGKIKDAFDRNPGLQNLLLDDFFKSAVENCQDSWRRAI     410
Bovine                          WTLNYGGIALMWRGGCIIRSVFLGKIKDAFDRNPGLQNLLLDDFFKSAVENCQDSWRRAI     410
Dog                             WTLNYGAIALMWRGGCIIRSVFLGKIKDAFDRNPQLQNLLLDDFFKSAVENCQDSWRRAV     410
Pig                             WSLNYGGIALM--------CVFLGKIKDAFDRNPGLQNLLLDDFFKSAVEDCQDSWRRAV     402
Giant_panda                     WTLNYGGVALMWRGGCIIRSVFLGKIKDAFDRNPQLQNLLLDDFFKSAVEDCQGSWRRAV     409
Horse                           WTLNYGGIALMWRGGCIIRSVFLGKIKDAFDRNPELENLLLDDFFRSAVESCQDSWRRAV     410
Atlantic_salmon                 WSLNYGAIALMWRGGCIIRSVFLGKIKEAFDRDAELQNLLLDTFFSNAVQDCQDSWRRTV     410
Osmerus_mordax                  WSLNYGAIALMWRGGCIIRSVFLGKIKEAFDRDFELQSLLLDNFFSNAVQDCQDSWRRTI     410
Black_porgy                     WSLNYGAIALMWRGGCIIRSVFLGKIKEAFDRDAELQNLLLDTFFSNAVQDCQESWRRAV     410
Anthurium_amnicola              WDLNNAGIALMWRGGCIIRSAFLGDIKNAYTNNPNLENLLFDPFFKKAIDDAQDSWRRVV     411
Chlamydomonas_reinhardtii       WNVDLGGLARIWKGGCIIRAQFLDRIRVAYERNPELPSLLVDPDFAKELTAAEASWRRVA     415
Zebrafish                       WSLNYGAIALMWRGGCIIRSVFLGKIKEAFDRNPELQSLLLDSFFSKAVQDCQDSWRRVV     410
Caenorhabditis_elegans          WNLNFGAIALMWRGGCIIRSRFLGDIEHAFQKNKQLSNLLLDDFFTKAITEAQDSWRVVV     411
Drosophila_melanogaster         WRLNYGGIALMWRGGCIIRSVFLGNIKDAYTSQPELSNLLLDDFFKKAIERGQDSWREVV     408
Yeast                           WKLNNPAIALMWRGGCIIRSVFLAEITKAYRDDPDLENLLFNEFFASAVTKAQSGWRRTI     412
                                * ::  .:* :        . **  *  *:  :  * .**.:  * . :   : .** .

Human                           STGVQAGIPMPCFTTALSFYDGYRHEMLPASLIQAQRDYFGAHTYELLAKP-------GQ     463
Rat                             STGVQAGIPMPCFTTALSFYDGYRHEMLPANLIQAQRDYFGAHTYELLSKP-------GE     463
Mouse                           STGVQAGIPMPCFTTALSFYDGYRHEMLPANLIQAQRDYFGAHTYELLTKP-------GE     463
Sheep                           STGVQAGIPMPCFTTALSFYDGYRHAMLPANLIQAQRDYFGAHTYELLAKP-------GQ     463
Bovine                          STGVQAGIPMPCFTTALSFYDGYRHEMLPANLIQAQRDYFGAHTYELLAKP-------GQ     463
Dog                             STGVQAGVPMPCFTTALSFYDGYRHEMLPANLIQAQRDYFGAHTYELLAKP-------GQ     463
Pig                             STGVQTGIPMPCFTTALSFYDGYRHEMLPANLIQAQRDYFGAHTYELLAKP-------GH     455
Giant_panda                     STGVQAGIPMPCFTTALSFYDGYRHEMLPANLIQAQRDYFGAHTYELLAKP-------GQ     462
Horse                           STGVQMGIPMPCFTTALSFYDGYRHEMLPANLIQAQRDYFGAHTYELLAKP-------GQ     463
Atlantic_salmon                 SIGVQQGIPMPCFTTALSFYDGYRHDMLPANLLQAQRDYFGAHTYELLSNP-------GK     463
Osmerus_mordax                  STGVQQGIPMPCFTTALSFYDGYRHGMLPANLLQAQRDYFGAHTYELLSNP-------GT     463
Black_porgy                     STGVQHGIPMPCFTTALSFYDGYRHEKLPANLLQAQRDYFGAHTYELLSNP-------GH     463
Anthurium_amnicola              AESAILGIPTPALSTALSFYDGYRHERLPANLLQAQRDYFGAHTFRYLDEYTDEKHPKDT     471
Chlamydomonas_reinhardtii       ALSITHGVPIPSMTSSLGYFDTYRRERLPANLVQAQRDFFGSHTYQRFDKE--------G     467
Zebrafish                       STGVQQGIPMPSFTTALSFYDGYRHEMLPANLLQAQRDYFGAHTYELLSNP-------GT     463
Caenorhabditis_elegans          CAAVRLGIPVPAFSSALAFYDGYTSEVVPANLLQAQRDYFGAHTYELLAKP-------GT     464
Drosophila_melanogaster         ANAFRWGIPVPALSTALSFYDGYRTAKLPANLLQAQRDYFGAHTYELLGQE-------GQ     461
Yeast                           ALAATYGIPTPAFSTALAFYDGYRSERLPANLLQAQRDYFGAHTFRILPECASAHLPVDK     472
                                . .   *:* *.::::*.::* *    :**.*:*****:**:**:. : :

Human                           FIHTNWTGHGGTVSSS---SYNA  483
Rat                             FIHTNWTGHGGSVSSS---SYNA  483
Mouse                           FIHTNWTGHGGSVSSS---SYNA  483
Sheep                           FIHTNWTGHGGSVSSS---SYNA  483
Bovine                          FIHTNWTGHGGSVSSS---SYNA  483
Dog                             FIHTNWTGHGGSVSSS---SYNA  483
Pig                             FVHTNWTGHGGSVSSS---SYNA  475
Giant_panda                     FIHTNWTGHGGSVSSS---SYNA  482
Horse                           FIHTNWTGHGGSVSSS---SYNA  483
Atlantic_salmon                 YIHTNWTGHGGNVSSS---SYNA  483
Osmerus_mordax                  HIHTNWTGHGGNVSSS---SYNA  483
Black_porgy                     FIHTNWTGHGGNVSSS---SYNA  483
Anthurium_amnicola              DVHINWTGHGGNVSSS---AYLA  491
Chlamydomonas_reinhardtii       WYHTVWDETFGSADSITTSGYVV  490
Zebrafish                       FIHTNWTGHGGKVSSS---SYNA  483
Caenorhabditis_elegans          WVHTNWTGTGGRVTSN---AYNA  484
Drosophila_melanogaster         FHHTNWTGTGGNVSAS---TYQA  481
Yeast                           DIHINWTGHGGNISSS---TYQA  492
                                  *  *    *   :     * .
```

Supplementary Fig. 1, related to Fig. 1

a, The peptides cover 92.55% of 6PGD sequences, and 6PGD pY481 was the only phosphorylation site detected in 6PGD (top panel). The presence of the b_{33}^{3+} at 1183.22704 and the b_{34}^{3+} at 1264.23692 indicates that the Y481 residue is phosphorylation modified (bottom panel).

b, GSC11 cells stably expressing Flag-6PGD WT or Y481F were treated with or without EGF (100 $ng \cdot ml^{-1}$) for 30 min.

c, GSC11 cells stably expressing Flag-6PGD WT or Y481F were treated with EGF (100 $ng \cdot ml^{-1}$) for 30 min.

d, Sequence alignment of full length 6PGD among indicated species.

e-h, Flag-6PGD WT or Y481F were immunoprecipitated from U251/EGFR cells or GSC11 cells with or without EGF (100 $ng \cdot ml^{-1}$) treatment for 30 min. The enzymatic activities of immunoprecipitated Flag-6PGD proteins were examined **(e, g)**. Statistical analyses of 6PGD activities **(f, h)**. The activity of 6PGD was normalized against protein amounts. 6PGD WT activity without EGF treatment was normalized to 1.0. Data represent the mean ± SD of normalized 6PGD activities. Student’s t test (unpaired, two-tailed), **p<0.01; n.s.: not significant

Immunoprecipitation and immunoblotting analyses were performed with the indicated antibodies. Data are representative of at least three independent experiments. **(b, c, f, h)** Source data are provided as a Source Data file.

Supplementary Fig. 2

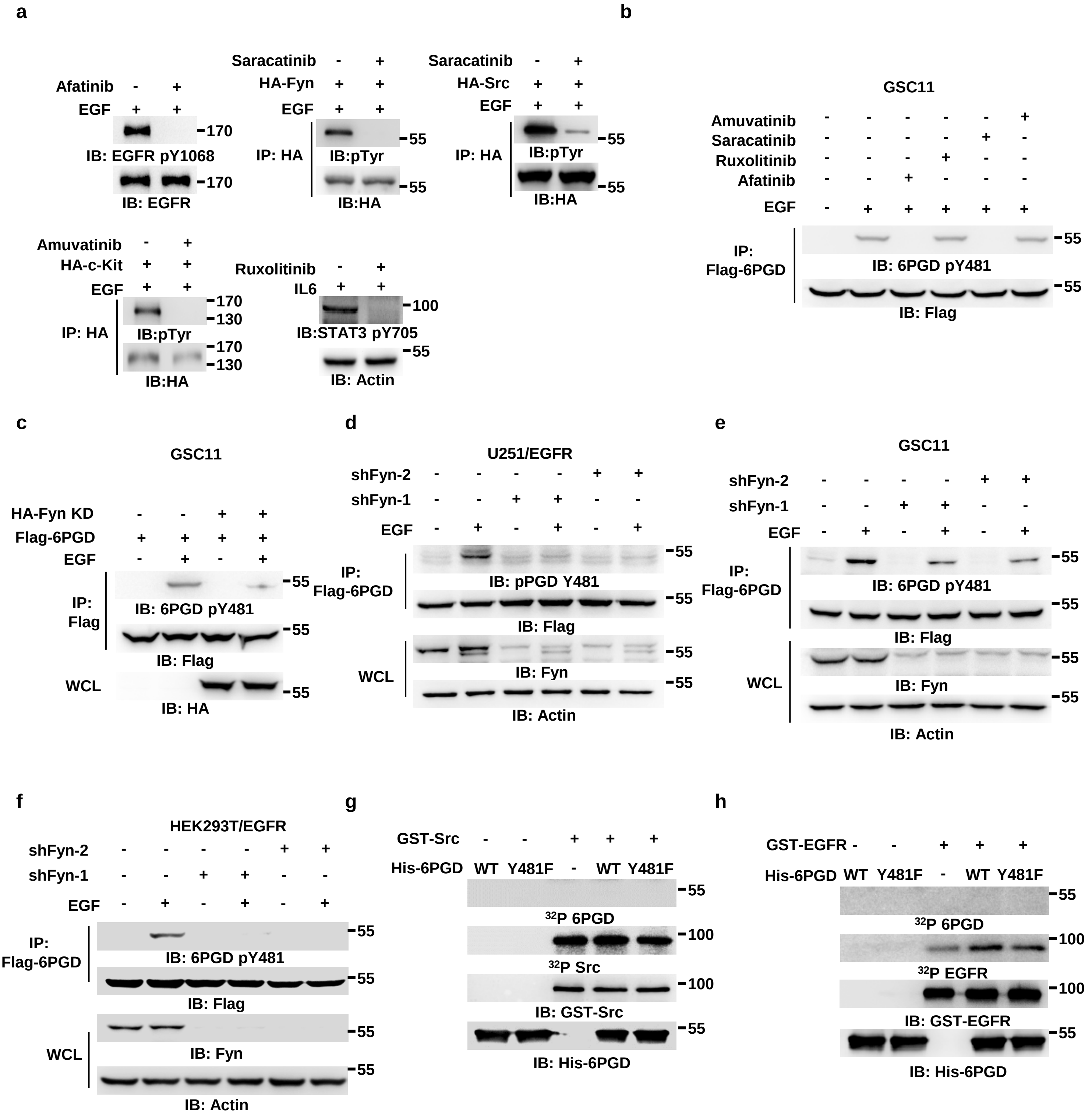

Supplementary Fig. 2, related to Fig. 2

a, HEK293T/EGFR cells were transfected with HA-Src, HA-Fyn or HA-c-Kit and then pretreated with EGFR inhibitor Afatinib (1 μM), Src family kinases inhibitor Saracatinib (1 μM) or c-Kit kinase inhibitor Amuvatinib (10 μM) for 3 hrs, followed by EGF treatment (100 $ng \cdot ml^{-1}$) for 30 min. U87 cells were pretreated with or without Ruxolitinib (1 μM) for 3 hrs, followed by the treatment of IL6 for 1 hr.

b, GSC11 cells stably expressing Flag-6PGD were pretreated with Afatinib (1 μM), Ruxolitinib (1 μM), Saracatinib (1 μM) or Amuvatinib (10 μM) for 3 hrs and then treated with EGF (100 $ng \cdot ml^{-1}$) for 30 min.

c, GSC11 cells stably expressing Flag-6PGD were infected with or without the lentivirus expressing a kinase-dead HA-Fyn (KD). These cells were then treated with or without EGF (100 $ng \cdot ml^{-1}$) for 30 min.

d-f, U251/EGFR cells **(d),** GSC11 cells **(e)** or HEK293T/EGFR cells **(f)** were infected with or without the lentivirus expressing shNT or two shRNA sequences against Fyn (shFyn-1 and shFyn-2). These cells were then treated with or without EGF (100 $ng \cdot ml^{-1}$) for 30 min as indicated.

g,h, *In vitro* kinase assays were carried out with purified recombinant His-6PGD WT or Y481F and commercial purchased recombinant GST-EGFR **(g)** or GST-Src **(h).**

Immunoprecipitation and immunoblotting analyses were performed with the indicated antibodies. Data are representative of at least three independent experiments. Source data are provided as a Source Data file.

Supplementary Fig. 3

a

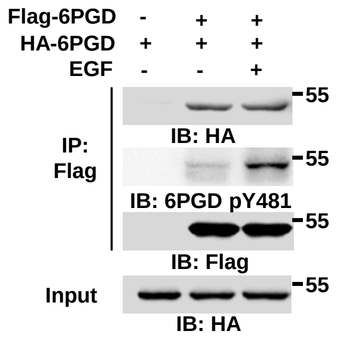

b

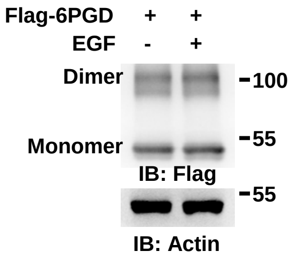

c

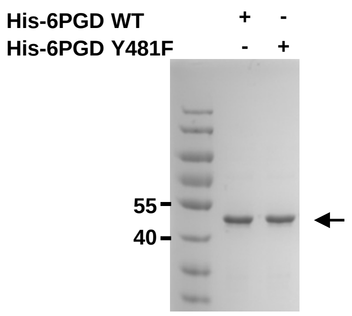

d

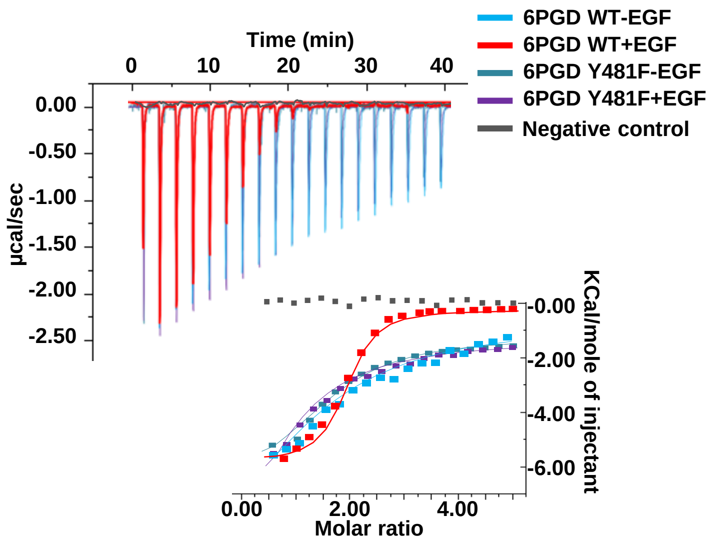

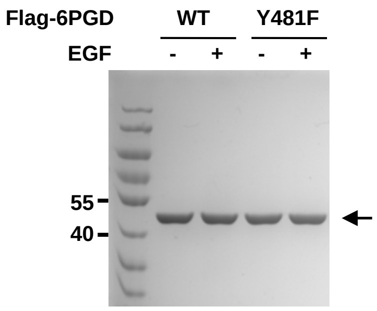

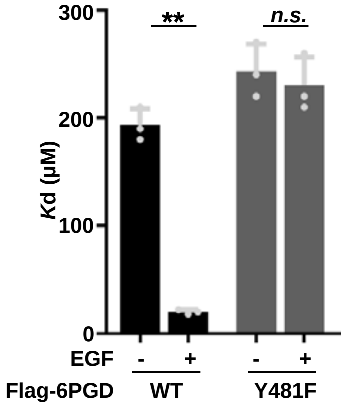

e

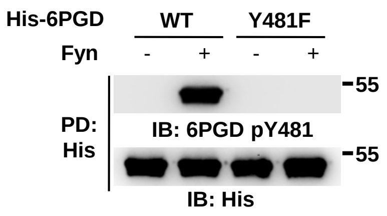

Supplementary Fig. 3, related to Fig. 3

a, U87/EGFR cells were transiently transfected with Flag-6PGD and HA-6PGD. The cells were treated with or without EGF (100 ng·ml^{-1}) for 30 min.

b, U87/EGFR cells stably expressing Flag-6PGD were treated with or without EGF (100 ng·ml^{-1}) for 30 min. Cell lysates were mixed with glutaraldehyde to the final concentration of 0.025% for 15 min.

c, The purities of bacterial purified recombinant His-6PGD WT and Y481F were verified by Coomassie blue staining (top panel). HEK293T/EGFR cells transfected with 6PGD WT and Y481F were treated with or without EGF (100 ng·ml^{-1}) for 30 min. The purities of immunoprecipitated 6PGD variants were verified by Coomassie blue staining (bottom panel).

d, HEK293T/EGFR cells transfected with 6PGD WT or Y481F were treated with or without EGF (100 ng·ml^{-1}) for 30 min. ITC assays were performed with immunoprecipitated 6PGD variants (0.05 mM) and $NADP^+$ (1 mM) (top panel). Flag peptides were used as negative control. Statistical analyses of *K*d values of 6PGD variants for $NADP^+$ were presented (bottom panel). Data represent the mean ± SD of three independent experiments. Student's t test (unpaired, two-tailed), **p<0.01; n.s.: not significant

e, *In vitro* kinase assays were performed by incubating bacterial purified recombinant His-6PGD WT or Y481F with or without active recombinant Fyn.

Immunoprecipitation and immunoblotting analyses were performed with the indicated antibodies. Data are representative of at least three independent experiments. Source data are provided as a Source Data file.

Supplementary Fig. 4

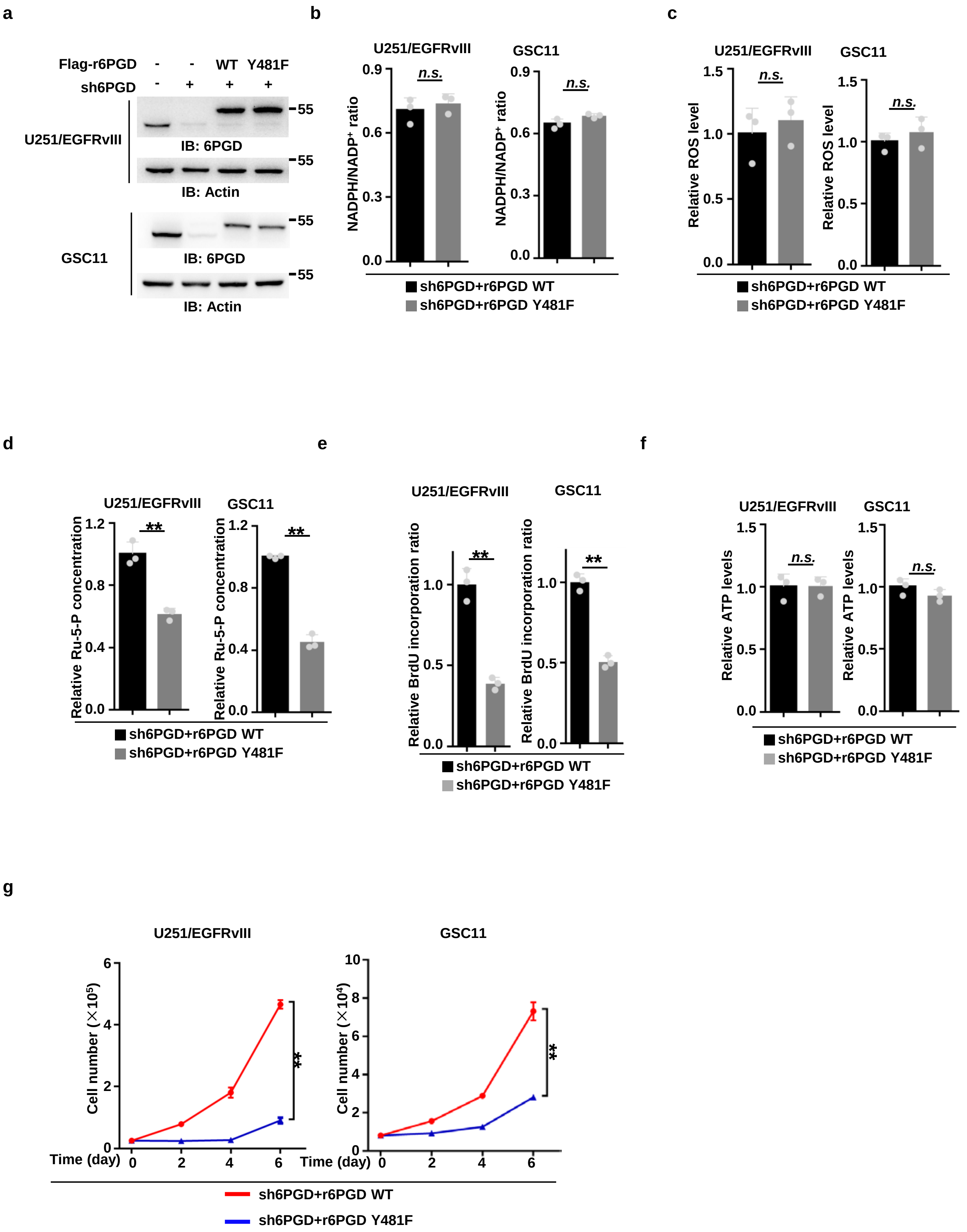

Supplementary Fig. 4, related to Fig. 4

a, U251/EGFRvIII cells or GSC11 cells stably expressing luciferase were depleted of endogenous 6PGD and reconstituted with the expression of r6PGD WT or Y481F. The expression of 6PGD was examined using immunoblotting assays. Data are representative of at least three independent experiments.

b-f, $NADP^+$/NADPH ratio **(b)**, ROS levels **(c)**, cellular Ru-5-P level **(d),** BrdU incorporation ratio **(e)** and cellular ATP level **(f)** were measured in these cells generated in **(a)**.

g, Cell proliferation was determined in these cells generated in **(a)**.

(b-g) Data represent the mean ± SD of three independent experiments. Student's t test (unpaired, two-tailed), **p<0.01; n.s.: not significant. Source data are provided as a Source Data file.

Supplementary Fig. 5

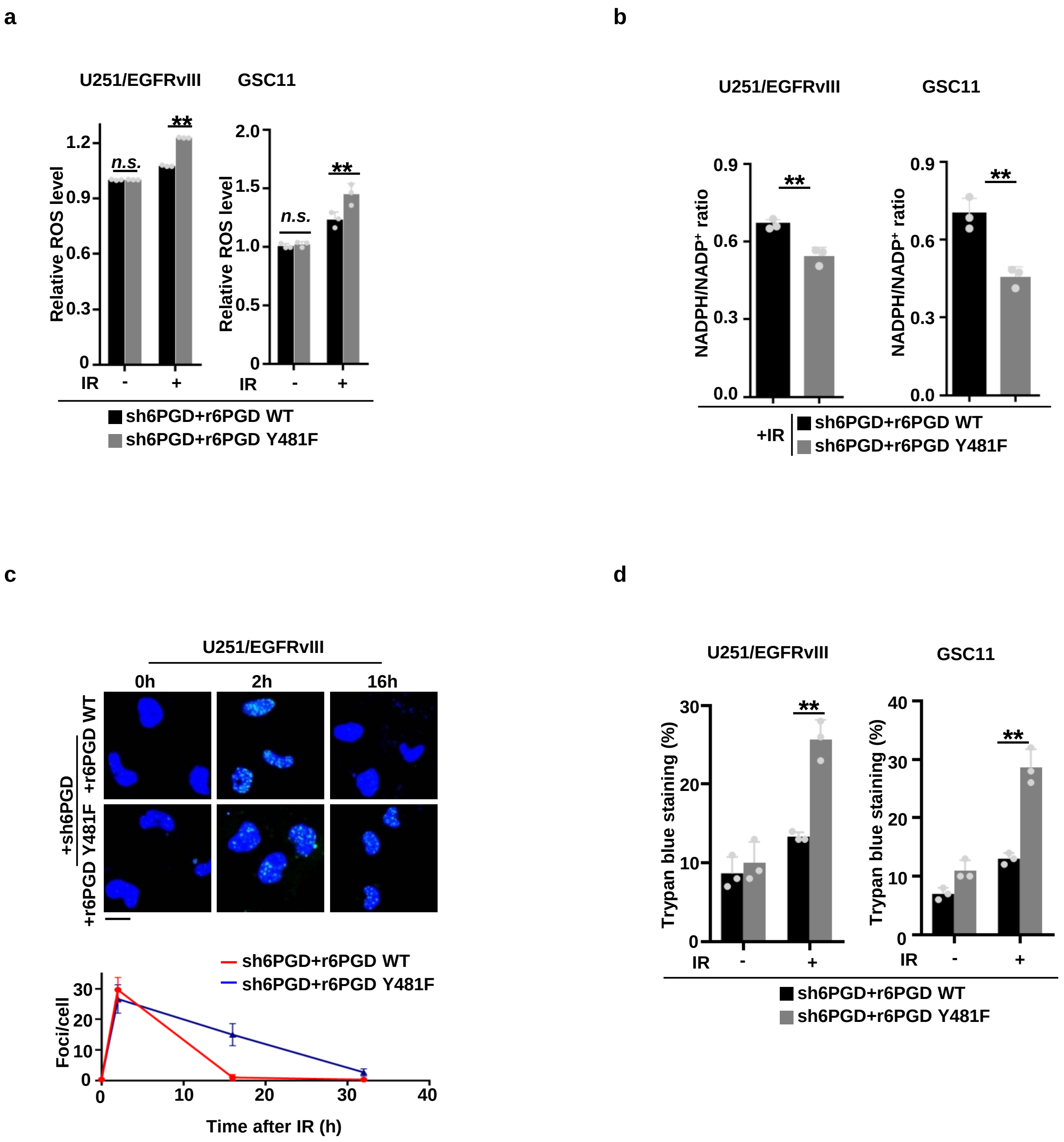

Supplementary Fig. 5, related to Fig. 5

a,b, U251/EGFRvIII cells or GSC11 cells stably expressing luciferase were depleted of endogenous 6PGD and reconstituted with the expression of r6PGD WT or Y481F. These cells were treated with or without X-ray radiation (10 Gy) and cultured for 8 hrs. ROS levels were measured using ROS assay kit **(a)**. NADPH/$NADP^+$ ratio were determined using $NADP^+$/NADPH quantitation kit **(b)**.

c, These genetically-modified U251/EGFRvIII cells generated in **(a)** were subjected to X-ray radiation (2 Gy) and cultured for indicated times. Immunofluorescence staining was performed using anti-γH2AX antibody. Scale bar, 20 μm

d, These cells generated in **(a)** were subjected to X-ray radiation (10 Gy) and cultured for 48 hrs. Cell death was determined using trypan blue staining.

Data represent the mean ± SD of three independent experiments. Student's t test (unpaired, two-tailed), $^{**}p<0.01$; n.s.: not significant. Source data are provided as a Source Data file.

Supplementary Fig. 6

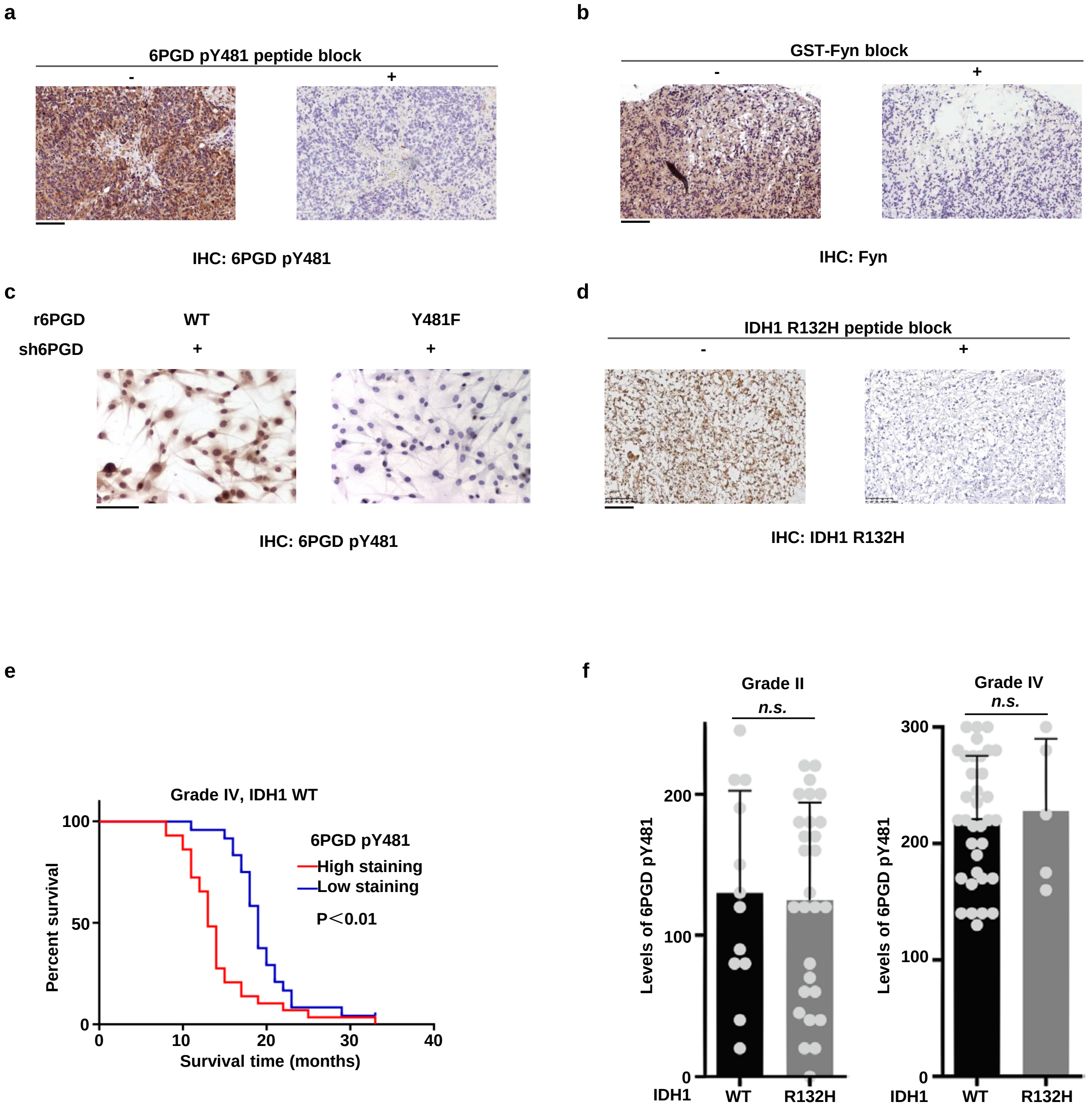

Supplementary Fig. 6, related to Fig. 6

a,b, Validation of the specificities of anti-6PGD pY481 or anti-Fyn antibody. IHC analyses of 6PGD pY481 or Fyn were performed in human GBM tissues with the indicated antibodies in the presence or absence of specific blocking peptides or proteins. Scale bar, 100 μm

c, Validation of the specificity of anti-6PGD pY481 antibody. IHC analyses of 6PGD pY481 were performed in endogenous 6PGD-depleted U87/EGFRvIII cells with rescued expression of r6PGD WT or Y481F. Scale bar, 100 μm

d, Validation of the specificity of anti-IDH1 R132H antibody. IHC analyses of IDH1 R132H were performed in human GBM tissues with this antibody in the presence or absence of specific blocking peptides. Scale bar, 100 μm

e, Survival durations of 53 GBM (IDH WT) patients with low (0-170 staining scores, blue curve) versus high (175-300 staining scores, red curve) 6PGD pY481 levels (low, 23 patients; high, 30 patients) were compared. Landmark represents censored (alive at last clinical follow-up) patients. Log-rank test, $p<0.01$.

f, IHC analyses of 6PGD pY481 or IDH1 R132H was performed in 40 diffuse astrocytoma (WHO grade II) specimens and 40 GBM (WHO grade IV) specimens using anti-6PGD pY481 and anti-IDH1 R132H antibody. IHC scores of 6PGD pY481 were compared between the low grade glioma patients with IDH1 WT (n=12) and the low grade glioma patients with IDH1 R132H (n=28) (left panel) or between the high grade GBM patients with IDH1 WT (n=35) and the high grade GBM patients with IDH1 R132H (n=5). Student's t-test (unpaired, two tailed), n.s.: not significant.

(e, f) Source data are provided as a Source Data file.

Supplementary Table 1. Parameters for LC/MS/MS analysis

**Compounds**	**Transition (m/z)**	**DP (V)**	**CE (eV)**	**Retention Time (min)**
G-6-P/F-6-P-m0, …, m6	259>97, …, 265>97	-50	-22	9.83
R-5-P-m0, …, m5	229>97, …, 234>97	-80	-19	9.73
Ru-5-P-m0, …, m5	229>97, …, 234>97	-80	-19	9.74
S-7-P-m0, …, m7	289>97, …, 296>97	-80	-20	10.22
glucose-m0, …, m6	239>179, …, 245>185	-50	-11	6.75
