# Supplementary material for: Tyrosine phosphorylation activates 6-phosphogluconate dehydrogenase and promotes tumor growth and radiation resistance
Source: Nat Commun. 2019 Mar 1;10:991. doi: 10.1038/s41467-019-08921-8 (PMC6397164; doi:10.1038/s41467-019-08921-8)
Supplement: Supplementary file 3 — Reporting Summary [file 41467_2019_8921_MOESM3_ESM.pdf]

nature research

Corresponding author(s): Weiwei Yang
Last updated by author(s): Jan 14, 2019

Reporting Summary

Nature Research wishes to improve the reproducibility of the work that we publish. This form provides structure for consistency and transparency in reporting. For further information on Nature Research policies, see Authors & Referees and the Editorial Policy Checklist.

Statistics

For all statistical analyses, confirm that the following items are present in the figure legend, table legend, main text, or Methods section.

n/a	Confirmed	
☐	☒	The exact sample size (*n*) for each experimental group/condition, given as a discrete number and unit of measurement
☐	☒	A statement on whether measurements were taken from distinct samples or whether the same sample was measured repeatedly
☐	☒	The statistical test(s) used AND whether they are one- or two-sided<br>*Only common tests should be described solely by name; describe more complex techniques in the Methods section.*
☒	☐	A description of all covariates tested
☒	☐	A description of any assumptions or corrections, such as tests of normality and adjustment for multiple comparisons
☐	☒	A full description of the statistical parameters including central tendency (e.g. means) or other basic estimates (e.g. regression coefficient) AND variation (e.g. standard deviation) or associated estimates of uncertainty (e.g. confidence intervals)
☒	☐	For null hypothesis testing, the test statistic (e.g. *F*, *t*, *r*) with confidence intervals, effect sizes, degrees of freedom and *P* value noted<br>*Give P values as exact values whenever suitable.*
☒	☐	For Bayesian analysis, information on the choice of priors and Markov chain Monte Carlo settings
☒	☐	For hierarchical and complex designs, identification of the appropriate level for tests and full reporting of outcomes
☐	☒	Estimates of effect sizes (e.g. Cohen's *d*, Pearson's *r*), indicating how they were calculated

Our web collection on statistics for biologists contains articles on many of the points above.

Software and code

Policy information about availability of computer code

Data collection	Graph Pad 6.0
Data analysis	Graph Pad 6.0

For manuscripts utilizing custom algorithms or software that are central to the research but not yet described in published literature, software must be made available to editors/reviewers. We strongly encourage code deposition in a community repository (e.g. GitHub). See the Nature Research guidelines for submitting code & software for further information.

Data

Policy information about availability of data

All manuscripts must include a data availability statement. This statement should provide the following information, where applicable:

- Accession codes, unique identifiers, or web links for publicly available datasets
- A list of figures that have associated raw data
- A description of any restrictions on data availability

All data supporting the conclusions are available from the authors on reasonable request.

Field-specific reporting

Please select the one below that is the best fit for your research. If you are not sure, read the appropriate sections before making your selection.

☒ Life sciences ☐ Behavioural & social sciences ☐ Ecological, evolutionary & environmental sciences

For a reference copy of the document with all sections, see nature.com/documents/nr-reporting-summary-flat.pdf

Life sciences study design

All studies must disclose on these points even when the disclosure is negative.

Sample size	The chosen sample size are based on the numbers used for previous publications, which is most optimal to generate statistically significant results.
Data exclusions	No samples or animals were excluded from the analyses.
Replication	All data presented reflect findings that were reproducible.
Randomization	The samples/cells were randomized to be examined. The mice were randomized to put into separate groups /cages for experiments.
Blinding	For all experiements, the investigators were divided into two groups. One group were blinded to allocation during experiments and outcome assessment.

Reporting for specific materials, systems and methods

We require information from authors about some types of materials, experimental systems and methods used in many studies. Here, indicate whether each material, system or method listed is relevant to your study. If you are not sure if a list item applies to your research, read the appropriate section before selecting a response.

Materials & experimental systems

n/a	Involved in the study
☐	☒ Antibodies
☐	☒ Eukaryotic cell lines
☒	☐ Palaeontology
☐	☒ Animals and other organisms
☐	☒ Human research participants
☒	☐ Clinical data

Methods

n/a	Involved in the study
☒	☐ ChIP-seq
☒	☐ Flow cytometry
☒	☐ MRI-based neuroimaging

Antibodies

Antibodies used	Mouse monoclonal antibodies against Flag (1:5000, F3165) and anti-Flag M2 affinity gel (A2220) were purchased from Sigma (St. Louis, MO, USA). Rabbit monoclonal antibodies against HA (1:3000, 3724S), β-actin (1:3000, 3700S), phospho-STAT3 Y705 (1:1000, 9145) and phospho-tyrosine (pTyr) (1:2000, 8954S), and rabbit polyclonal antibodies against Fyn (1:3000 for IB, 1:200 for IHC, 4023S) and phospho-EGFR Y1068 (1:1000, 2234) were obtained from Cell Signaling Technology (Danvers, MA, USA). Rabbit polyclonal antibodies against 6PGD (1:3000, 14718-1-AP), GST (1:5000, 10000-0-AP) and Flag (1:5000, 20543-1-AP), and mouse monoclonal antibody against His (1:5000, 66005-1-Ig) were purchased from Proteintech (Wuhan, China). Rabbit polyclonal antibody against phospho-serine (pSer) (1:1000, AB1603) was purchased from Millipore (Darmstadt, Germany). Mouse monoclonal antibody against human IDH1-R132H (1:100, D299-3) was purchased from Medical & Biological Laboratories (Tokyo, Japan). Mouse monoclonal antibody against phospho-threonine (pThr) (1:1000, sc-5267) was purchased from Santa cruz biotechnology (CA, USA). The custom-designed rabbit polyclonal antibody against phospho-6PGD Y481 [CTNWTGHGGTVSSSS(pY)NA] (1:1000 for IB, 1:100 for IHC) were obtained from Abclonal Technology (Wuhan, China).
Validation	The antibodies for IHC studies were validated in Supplementary Figure 6. All other commercial antibodies were validated by manufacturers.

Eukaryotic cell lines

Policy information about cell lines

Cell line source(s)	U87 (TCHu138), U251 (TCHu58) and HEK293T (GNHu17) cells were obtained from the cell library of the Chinese Academy of Sciences. GSC11 cells are primary human GBM cells from MD Anderson.
Authentication	Cells were authenticated using the short tandem repeat (STR) method.
Mycoplasma contamination	All cell lines were routinely tested negative for mycoplasma contamination.
Commonly misidentified lines (See ICLAC register)	No commonly misidentified cell lines were used.

Animals and other organisms

Policy information about studies involving animals; ARRIVE guidelines recommended for reporting animal research

Laboratory animals	athymic nude mice, female, 8 weeks.
Wild animals	The study did not involve wild animals.
Field-collected samples	The study did not involve samples collected from the field.
Ethics oversight	The use of mice was following ethical regulations and was approved by the institutional review board at the Institute of Biochemistry and Cell Biology.

Note that full information on the approval of the study protocol must also be provided in the manuscript.

Human research participants

Policy information about studies involving human research participants

Population characteristics	Human research participants includes male and female, all of whom received standard adjuvant radiotherapy and chemotherapy after surgery.
Recruitment	N/A
Ethics oversight	The use of human brain tumor specimens and the database was approved by the Institutional Review Board at XinHua Hospital School of Medicine and the institutional review board at First Affiliated Hospital of Wenzhou Medical College. Informed consent was obtained from all patients.

Note that full information on the approval of the study protocol must also be provided in the manuscript.
